# Validation of targeted next-generation sequencing panels in a cohort of Polish patients with epilepsy: assessing variable performance across clinical endophenotypes and uncovering novel genetic variants

**DOI:** 10.3389/fneur.2023.1316933

**Published:** 2024-01-12

**Authors:** Magdalena Badura-Stronka, Katarzyna Wołyńska, Anna Winczewska-Wiktor, Justyna Marcinkowska, Dagmara Karolewska, Danuta Tomkowiak-Kędzia, Michał Piechota, Marta Przyborska, Natalia Kochalska, Barbara Steinborn

**Affiliations:** ^1^Chair and Department of Medical Genetics, Poznan University of Medical Sciences, Poznań, Poland; ^2^Centers for Medical Genetics GENESIS, Poznań, Poland; ^3^Chair and Department of Developmental Neurology, Poznan University of Medical Sciences, Poznań, Poland; ^4^Chair and Department of Informatics and Statistics, Poznan University of Medical Sciences, Poznań, Poland; ^5^Center for Child’s Health of Wielkopolska Region, Poznań, Poland

**Keywords:** next-generation sequencing, ILAE, epilepsy, genetics, yield, panels

## Abstract

**Introduction:**

Targeted Next-Generation Sequencing Panels (TNGSP) have become a standard in global clinical practice. Instead of questioning the necessity of next-generation sequencing in epilepsy patients, contemporary large-scale research focuses on factors such as the size of TNGSP, the comparative advantages of exome or genome-wide sequencing over TNGSP, and the impact of clinical, electrophysiological, and demographic variables on genetic test performance. This study aims to elucidate the demographic and clinical factors influencing the performance of TNGSP in 138 Polish patients with epilepsy, recognizing the pivotal role of genetic testing in guiding patient management and therapy.

**Methods:**

A retrospective analysis was conducted on patients from a genetic clinic in Poznań, Poland, who underwent commercial gene panel studies at Invitae Corporation (USA) between 2020 and 2022. Patient groups were defined based on the age of onset of the first epileptic seizures, seizure type, gender, fever dependence of seizures, presence of intellectual disability or developmental delay, abnormalities in MRI, and the presence of dysmorphic features or congenital malformations. Seizure classification followed the 2017 ILAE criteria.

**Results:**

Among the 138 patients, 30 (21.7%) exhibited a pathogenic or likely pathogenic variant, with a distribution of 20.7% in males and 22.5% in females. Diagnostic performance correlated with the patient’s age at the onset of the first seizure and the type of seizure. Predominant variants were identified in the *SCN1A, PRRT2, CDKL5, DEPDC5, TSC2*, and *SLC2A1* genes. Additionally, 12 genes (*CACNA1A, SCN2A, GRIN2A*, *KCNQ2, CHD2, DYNC1H1, NEXMIF, SCN1B, DDX3X, EEF1A2, NPRL3, UBE3A*) exhibited single instances of damage. Notably, novel variants were discovered in *DEPDC5, SCN1A, TSC2, CDKL5, NPRL3, DYNC1H1, CHD2*, and *DDX3X*.

**Discussion:**

Identified variants were present in genes previously recognized in both European and non-European populations. A thorough examination of Variants of Uncertain Significance (VUSs), specifically focusing on gene copy number changes, may unveil more extensive chromosomal aberrations. The relatively frequent occurrence of pathological variants in X chromosome-linked genes in girls warrants further investigation, challenging the prevailing notion of male predominance in X-linked epilepsy.

## Introduction

Physicians’ perception of the importance of genetic testing in patients with neurological and neurodevelopmental disorders has changed dramatically over the last two decades. In particular, this applies to epilepsy. Back in 2010, in the ILAE (International League Against Epilepsy) report on genetic testing in epilepsy, there was a statement based on the work from 2006 that monogenic epilepsy constitutes a “tiny” proportion of all epilepsy (1). It has been argued that the low rate of familial epilepsy suggests the multifactorial nature of the condition (1). The methods described at that time, used in diagnosing genetic causes of epilepsy, made it possible to determine the underlying cause in a few patients. These were variant detection screening methods, such as DHPLC (denaturing high-performance liquid chromatography), CSGE (conformation sensitive gel electrophoresis), DGGE (denaturing gradient gel electrophoresis), Sanger sequencing, Array Comparative Genomic Hybridization (array-CGH), fluorescent *in situ* hybridization (FISH), single nucleotide polymorphism arrays (SNP arrays), multiplex ligation-dependent probe amplification (MLPA), Southern blot analysis (1). These methods were tedious, long-lasting, requiring much laboratory work, and relatively expensive. At that time, they already allowed for a comprehensive examination of patients for small chromosomal changes, but the diagnosis of individual genes took months. Laboratories specialized in studying a few or a dozen genes and, with great effort, tested the gene-by-gene method of carefully selected patients with epilepsy.

With the advent of next-generation sequencing on the genetic testing market and the gradual reduction of testing costs, people with epilepsy were increasingly willing to undergo high-throughput genetic testing. Targeted Next-Generation Sequencing Panels (TNGSP) are now commonly used in everyday clinical practice worldwide. Commercial laboratories can furnish results from the analysis of a panel comprising 70 genes in 8,565 epilepsy patients. Additionally, there is a TNGSP study encompassing 89–133 genes, conducted on a substantial cohort of 2,008 adults with epilepsy (2, 3). The methodology of population studies, based on the analysis of 104 genes in the entire population of Scottish children with epilepsy diagnosed up to 3 years of age, impresses with its complexity (4). Not only is epilepsy caused by a single gene variant not uncommon, but they account for at least one-tenth of the causes of epilepsy (5–8). Common variants in multiple genes are still believed to be responsible for the majority of epilepsy cases. Complex inheritance explains the vast majority of idiopathic generalized epilepsies, such as childhood absence epilepsy, juvenile myoclonic epilepsy, juvenile absence epilepsy, and epilepsy with generalized tonic–clonic seizures alone (9). Additionally, multifactorial inheritance influences factors such as the age of first seizures, the severity of epilepsy, and the response to antiepileptic drugs (10, 11). Nevertheless, even in patients previously thought to have a multifactorial cause for their epilepsy, such as those with a structural cortical malformation, a monogenic cause is increasingly identified. Notably, variants in genes affecting the GATOR1 or MTOR pathway serve as prime examples of such monogenic causes (12, 13).

Every clinical geneticist now has Targeted Next-Generation Sequencing Panels at their fingertips, which shortens the time to establish a diagnosis of monogenic epilepsy to a few weeks or months. The identification of a monogenic cause of epilepsy refines the diagnosis and has far-reaching implications for treatment decisions, prognosis assessment, and participation in research initiatives aimed at advancing personalized medicine for epilepsy (2, 14). Some epilepsy medications may be less effective or even contraindicated based on the underlying genetic cause. Knowledge of the specific genetic mutation can help avoid the use of ineffective treatments, reducing the risk of adverse effects (2). Analyzes of the impact of genetic testing results on clinical decisions become more cautious over the years in estimating the positive effects of obtaining a diagnosis of monogenic epilepsy, which does not change the fact that the importance of such a diagnosis for genetic counseling and the possibility of providing more precise information to the family about the natural history of the disease is invaluable (15, 16). Large-scale researchers are no longer trying to prove that the use of next-generation sequencing tools in patients with epilepsy is essential but are investigating whether TNGSP size matters, investigating the superiority of exome or genome-wide sequencing over TNGSP, and determining what clinical electrophysiological and demographic factors affect the performance of genetic tests (6–8, 15–17).

This study aims to assess the variable performance of Targeted Next-Generation Sequencing Panels across different clinical endophenotypes in a cohort of 138 Polish patients with epilepsy.

## Methods

### Patients

The retrospective analysis included 138 patients of a genetic clinic in Poznań (Poland) who performed a commercial gene panel study at Invitae Corporation (USA) in 2020–2022. Patients were sent to our center by family doctors and child neurologists or presented without a doctor’s referral, which is also possible in Poland. The study was performed according to the actual review of the Declaration of Helsinki (18). As the study is a retrospective analysis, no major risks and burdens to the research subjects occurred. A precaution has been taken to protect the privacy of research subjects and the confidentiality of their personal information. All patients or caregivers gave their written consent for molecular tests.

Demographic data of the group covered by the analysis is presented in Table 1.

**Table 1:** Demographic and clinical characteristics of patients under study.

Demographic and clinical data		Percentage of patients in subgroups and demographic data for the entire group
Total number of patients		138 (100%)
Ethnicity		Caucasian (Polish)
Sex	Male	58 (42.03%)
	Female	80 (57.97%)
Age at testing (months)	Median [Q1; Q3]	79 [29.25; 151.5]
	Min; Max	0; 516
Age at first seizure	0–12 months	56 (40.6%)
	All >12 months	82 (59.4%)
	13–24 months	20 (14.49%)
	25 months–10 years	46 (33.3%)
	> 10 years	16 (11.59%)
ID/psychomotor retardation	Yes	45/115 (39.1%)
	No	70/115 (60.9%)
	NDA	23 (16.67%)
Category of epilepsy	Generalized	36 (26.09%)
	Focal	58 (42.03%)
	Epileptic and developmental encephalopathy	44 (31.88%)
Dysmorphic traits/MCA	Yes	28 (20.3%)
	No	110 (79.7%)
Fever sensitive seizures	Yes	27 (19.6%)
	No	111 (80.4%)

ID, intellectual disability; NDA, no data available; MCA, multiple congenital anomalies.

The patients under examination were aged from several weeks to 516 months at the time of the examination. The median age was 79 months. Boys and girls did not differ significantly in age (Mann–Whitney test, *p* = 0.48). The patients were categorized into different epilepsy endophenotypes for analysis, taking into consideration several factors. These factors included the age of onset of the first epileptic seizures, the type of epileptic seizures, gender, the association of seizures with fever, the presence of intellectual disability or developmental delay, the presence of dysmorphic features or congenital malformations, and the identification of abnormalities in magnetic resonance imaging (MRI) of the brain. This comprehensive approach allowed for a nuanced exploration of distinct subgroups within the patient population, enabling a more detailed examination of the relationship between genetic variants and specific clinical features associated with epilepsy.

In this study, patients were categorized into three groups based on the type of epilepsy they presented: those with focal seizures, those with generalized seizures, and those with developmental and epileptic encephalopathy (DEE). The classification criteria for seizures, as outlined in the 2017 International League Against Epilepsy (ILAE) classification, were employed (19). This classification approach is well-established and widely used in clinical settings, enhancing the comprehensibility and scalability of the study results for neurologists and other medical professionals. The categorization of patients into specific groups was carried out by a collaborative team comprising a clinical geneticist and a pediatric neurologist with expertise in epileptology. This multidisciplinary approach involved considering various factors, including the type of seizures experienced by the patients, the age of onset of seizures, findings from EEG recordings, and co-morbidities. Patients with GEFS+ phenotype were included in the group with generalized seizures. A group of patients with DEE had epilepsy early in life, with delayed psychomotor development caused by underlying etiology and poorly controlled seizures (19). Patients were qualified to the group with dysmorphic features by a clinical geneticist with experience in dysmorphology. By leveraging the insights of both a clinical geneticist and a pediatric neurologist specialized in epileptology, the team aimed to ensure a comprehensive and accurate classification of patients into distinct groups based on their clinical characteristics. In some patients, it was impossible to determine whether psychomotor development was normal due to their young age. The remaining patients were qualified for the group with intellectual disability/intellectual developmental disorder according to the Diagnostic and Statistical Manual of Mental Disorders (DSM-5) (20). The yield was defined as the percentage of patients with pathogenic/likely pathogenic variants in genes in the patients included in the disease categories.

### Genetic testing and variant interpretation

Genomic DNA was obtained from the submitted samples (blood or buccal swab) and epilepsy-related genes from a multigene panel were targeted and sequenced via a short-read next-generation sequencing (NGS) assay as previously published (21). Each gene on Invitae’s NGS panels is targeted with oligonucleotide baits (Agilent Technologies, Santa Clara, CA) to capture exons, the 10–20 bases of introns flanking exons, and several sequences in non-coding sequences of interest. The test obtained an average of 350x depth-of-sequence read coverage (21).

A bioinformatics pipeline aligned sequencing reads and used community standard and custom algorithms that identified single nucleotide variants, small insertions or deletions (indels), large indels, structural variants, and exon-level copy number variants (CNVs), as described previously (21–23). The gene panel included 104–304 genes and, in most patients, 290 genes or more (Supplementary Table S2). The content of genetic testing panels related to epilepsy has been updated and expanded over time by Invitae as new genes were discovered. In two cases, with a clinical suspicion of tuberous sclerosis, a smaller panel of 104 and 150 genes was used, aiming to analyze mainly the *TSC1* and *TSC2* genes.

Variants identified by the bioinformatics pipeline were analyzed by Sherloc, a proprietary, points-based framework based on the joint consensus guidelines from the American College of Medical Genetics and Genomics and the Association for Molecular Pathology (24). Multiple evidence types are considered during variant classification, including population data (from Exac and GnomAD database), the variant type, clinical observations, experimental studies, and indirect and computational methods (considered evidence types for variants defined as pathogenic and likely pathogenic can be found in Supplementary Table S1). Based on the evidence above, variants were classified as benign or likely benign (B/LB), variant(s) of uncertain significance (VUS), or pathogenic or likely pathogenic (P/LP). Once a variant is classified in 1 case, subsequent cases in which the variant is observed are automatically assigned the same interpretation in the absence of new data (i.e., new publications or internal inheritance/clinical information). The report included variants classified as P/LP or VUS, while B/LB variants were not reported.

Confirmation of the presence and location of reportable variants was performed based on stringent criteria established by Invitae Corporation (1400 16th Street, San Francisco, CA 94103, #05D2040778), as needed, using one of several validated orthogonal approaches (22). After high-throughput sequencing using Illumina technology, the output reads were aligned to a reference sequence (genome build GRCh37; custom derivative of the RefSeq transcriptome) to identify the locations of exon junctions through the detection of split reads. The relative usage of exon junctions in a test specimen was assessed quantitatively and compared to that seen in control specimens. The Sherloc variant interpretation framework evaluated abnormal exon junction usage as evidence. When a whole gene deletion was found, chromosomal rearrangements were confirmed in cases where patients consented by MLPA, qPCR or array- CGH techniques. All reportable variants observed at Invitae were de-identified and deposited in the Clinvar database and are available for research studies.

Oligonucleotide array-CGH analysis was performed using Agilent SurePrint G3 CGH ISCA v2, 8x60K microarray. The data were analyzed using AgilentCytoGenomics 5.0.2.5 software, and GRCh37/hg19 build was used as reference genome assembly. The analysis was conducted with the following parameters: 0.10 Mb detection level, the filter 5 probes in line, and DLR spread <0.3.

A VUS or a single pathogenic or likely pathogenic variant in a gene for the autosomal recessive disease was considered non-diagnostic.

### Statistical analyses

Statistical analyses were carried out using PQStat Software v.1.8. Before conducting the statistical tests, continuous data were assessed for normality using the Shapiro–Wilk test. A significance level of *p*-value < 0.05 was predetermined. To compare the two groups, the Mann–Whitney rank test was employed.

Dunn’s *post-hoc* analysis with Bonferroni correction for ANOVA Kruskal-Wallis test was employed to compare multiple groups. Additionally, Fisher’s test explored potential dependencies or associations among the variables under investigation.

Logistic regression models were utilized to explore predictors for prediction and classification, focusing on specific variables of interest. Common clinical baseline parameters were considered as potential predictors in these models.

The logistic regression analysis yielded odds ratios (ORs) and their corresponding 95% confidence intervals (CIs) as results.

To identify the most appropriate model, we thoroughly compared the logistic regression models using the Akaike Information Criterion (AIC) and the Bayesian Information Criterion (BIC).

## Results

### Performance of targeted panel genetic testing in the entire group of Polish patients with epilepsy and in different epilepsy endophenotypes

**Figure 1:**
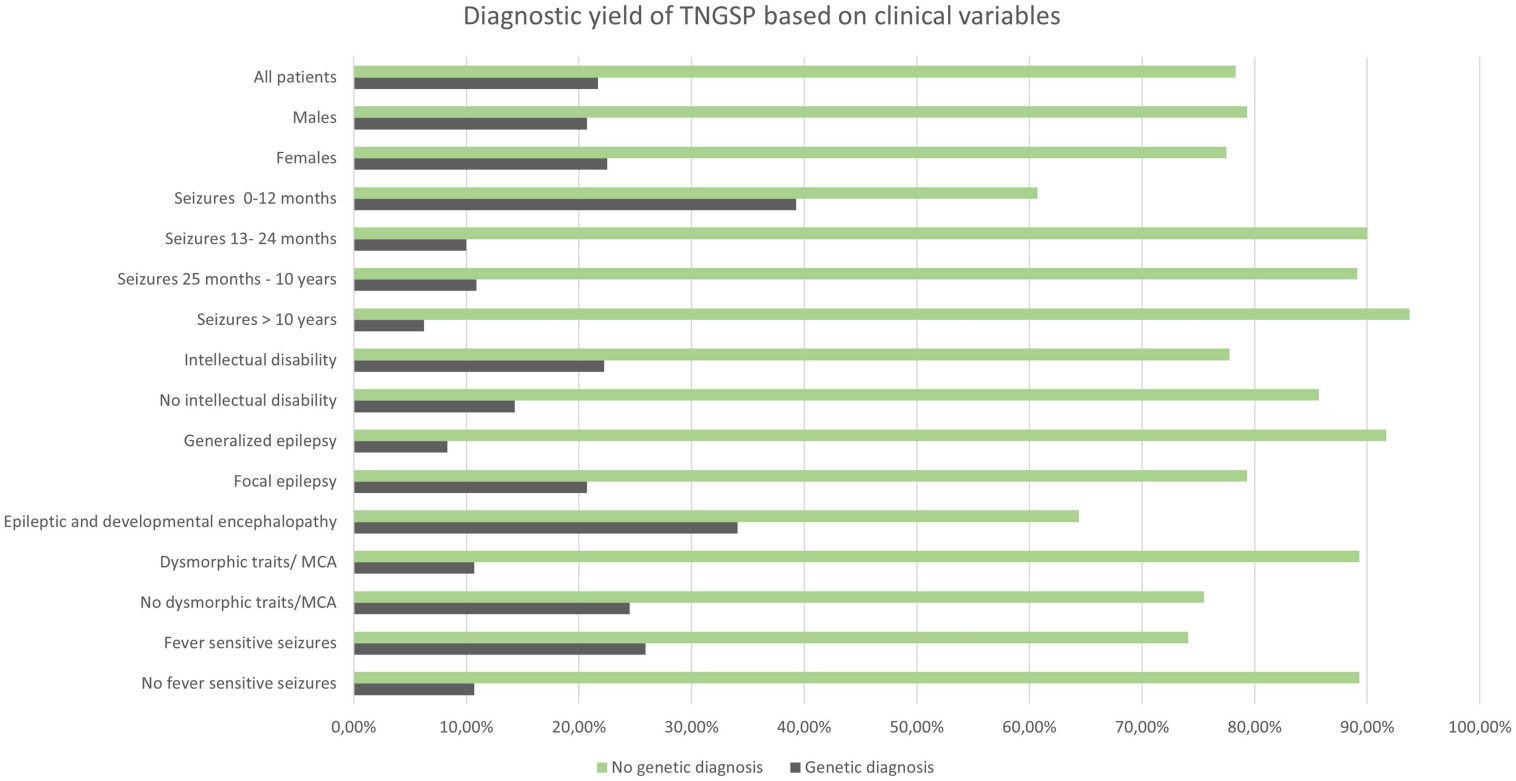
Diagnostic yield achieved with TNGSP in different epilepsy endophenotypes.

In 30 out of 138 patients from the study group, a pathogenic or likely pathogenic variant was found (21.7%) in 20.7% of males and 22.5% of females. Table 2 shows the diagnostic performance of the test in each clinical subcategory of patients. For most categories (sex, induction of seizures by fever, intellectual disability, presence of dysmorphic features and/or multiple congenital anomalies) the differences were statistically insignificant (Table 2; Figure 1).

**Table 2:** The yield of TNGSP in clinical subgroups of patients with epilepsy.

Demographic and clinical data		The yield of genetic testing in subgroups		
		Genetic diagnosis	No genetic diagnosis	All
Total number of patients		30 (21.7%)	108 (78.3%)	138
Sex	Male	12 (20.69%)	46 (79.31%)	58
	Female	18 (22.5%)	62 (77.5%)	80
Age at first seizure	0–12 months	22 (39.3%)	34 (60.7%)	56
	All >12 months	8 (9.8%)	74 (90.2%)	82
	13–24 months	2 (10%)	18 (90%)	20
	25 months–10 years	5 (10.9%)	41 (89.1%)	46
	>10 years	1 (6.25%)	15 (93.75%)	16
ID/intellectual developmental disorder	Yes	10 (22.22%)	35 (77.78%)	45
	No	10 (14.29%)	60 (85.71%)	70
Category of epilepsy	Generalized	3 (8.33%)	33 (91.67%)	36
	Focal	12 (20.69%)	46 (79.31%)	58
	Epileptic and developmental encephalopathy	15 (34.09%)	29 (64.4%)	44
Dysmorphic traits/MCA	Yes	3 (10.7%)	25 (89.3%)	28
	No	27 (24.5%)	83 (75.5%)	110
Fever sensitive seizures	Yes	7 (25.9%)	20 (74.1%)	27
	No	23 (10.7%)	88 (89.3%)	111

ID, intellectual disability; MCA, multiple congenital anomalies; statistically significant differences are marked with gray color: A statistically significant difference was found between diagnostic performance in patients with generalized epilepsy compared to patients with epileptic encephalopathies (*p* = 0.01817). Below the optimal age limit at 12 months (calculated from the ROC curve), it was nearly six times more likely (OR = 5.99) to detect the causative variant using a panel based on next-generation sequencing.

The diagnostic performance of the test was related to the patient’s age at the time of the first seizure.

Below the optimal age limit at 12 months (calculated from the ROC curve), it was nearly six times more likely (OR = 5.99) to detect the causative variant using a panel based on next-generation sequencing. Further logistic analysis (*p*-value < 0.000058) of the likelihood ratio test confirmed that the chances of detecting the causative variant decrease significantly (*p* = 0.003182) with each month of the child’s age: OR = 0.98 (0.96; 0.99).

The Fisher–Freeman–Halton test (*p* = 0.020108) confirmed that most often, in 34.09% of cases, the genetic cause was identified in patients with epileptic and developmental encephalopathy. A genetic cause was identified less frequently in patients with focal epilepsy (20.69%) and least frequently in patients with generalized epilepsy, according to the ILAE classification (8.33%). Using multiple comparisons with the Benjamini-Hochberg correction, no statistically significant difference was found between the efficiency of the method between patients with focal epilepsy and patients with generalized epilepsy, as well as between patients with focal epilepsy and patients with epileptic and developmental encephalopathy. A statistically significant difference was found between diagnostic performance in patients with generalized epilepsy compared to patients with epileptic encephalopathies (*p* = 0.01817).

### Diagnostic yield of TNGSP depending on MRI findings

We conducted an analysis of the diagnostic yield of targeted panel testing in a cohort of 94 patients for whom brain MRI data were available. The identified MRI abnormalities were categorized into two groups: those indicative of an environmental etiology for epilepsy (such as cerebral hemorrhage, extensive ischemic changes in hemispheres and basal ganglia) and those suggestive of a genetic origin (including significant brain atrophy, cortical dysplasia, changes typical for tuberous sclerosis, and hypoplasia of the corpus callosum). Among the patients, 64 exhibited either no MRI abnormalities or only minor ones (e.g., small arachnoid cyst, minimal white matter hyperintense changes in T2/FLAIR sequence, mild ventricular asymmetry). In 16 patients, the MRI findings pointed toward an environmental cause of epilepsy, while in 14 patients, a genetic basis was suggested.

**Figure 2:**
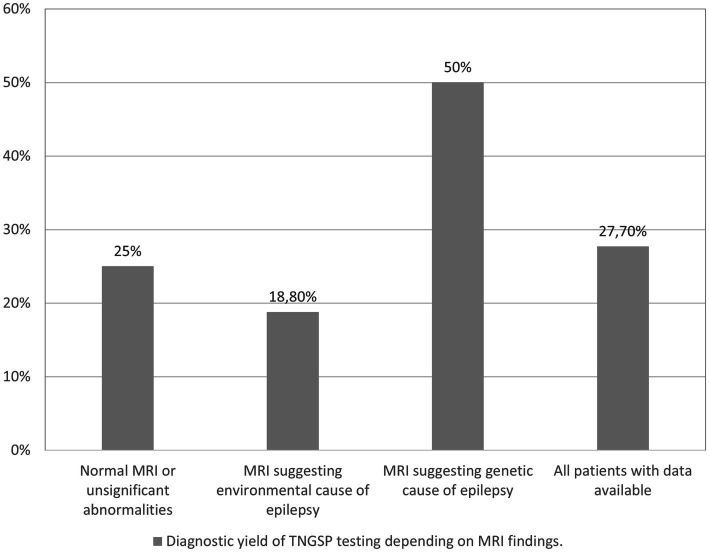
Diagnostic yield of TNGSP testing depending on MRI findings.

Figure 2 illustrates the diagnostic yield of genetic testing based on the MRI findings. The highest diagnostic yield was observed in patients with indications of a genetic cause on imaging, while the lowest yield was found in patients with indications of an environmental cause. However, it is noteworthy that these outcomes did not attain statistical significance.

### Likely pathogenic and pathogenic variants identified with TNGSP in our cohort

**Figure 3:**
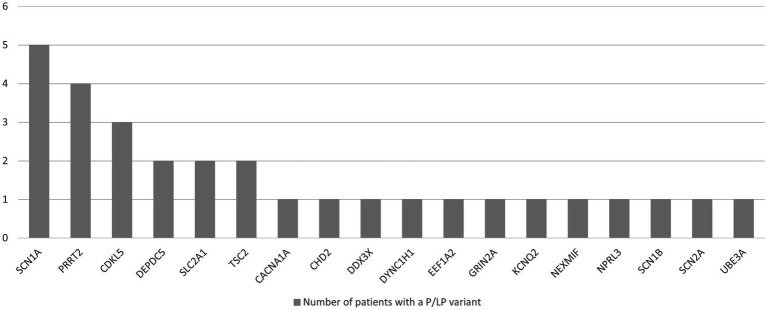
Number of patients with epilepsy with a likely pathogenic (LP) or pathogenic (P) variant in a particular gene, identified with TNGSP.

The most common variants were found in the *SCN1A*, *PRRT2*, *CDKL5*, *DEPDC5*, *TSC2*, and *SLC2A1* genes. The remaining 12 genes were damaged in single children (*CACNA1A, SCN2A, GRIN2A, KCNQ2, CHD2, DYNC1H1, NEXMIF, SCN1B, DDX3X, EEF1A2, NPRL3, UBE3A*) (Figure 3). Fifteen variants were missense, 8 nonsense, 2 splicing, and 5 deletions involving a part or the entire gene. We identified 9 novel variants in *DEPDC5, SCN1A, TSC2, CDKL5, NPRL3, CHD2, DYNC1H1,* and *DDX3X.*

In patient E122, with drug-resistant focal epilepsy, and normal brain MRI, we have identified a novel variant in the *DEPDC5* gene, a deletion of the entire coding sequence. Smaller deletions of several exons of the gene have been described previously (8 deletions in this gene, involving exons 28–38, 1–8, 2–3, 31–42, 40, 8–19) are included in the Human Gene Mutation Database (HGMD). According to Franklin loss of function variants in this gene are pathogenic. Described deletions caused focal epilepsy with variable loci, the same type of epilepsy as in our patient.

In a young boy experiencing fever-induced focal seizures since the age of 7 years, along with microcephaly and mild dysmorphic signs, we detected a novel variant in the *SCN1A* gene [c.5379del, p.(Glu1794Lysfs7)], Clinvar ID 1375417. This variant is expected to disrupt the last 216 amino acids of the SCN1A protein. Notably, this disruptive effect occurs within a region of the SCN1A protein where a previously identified variant [p.(Arg1922*), p.(Arg1882*)] has been established as pathogenic (25). Fukuma described these two variants in three children. In our patients the phenotype is milder than in reported patients, who had first seizures before the age of 1 year, moderately retarded psychomotor development, and myoclonic seizures (with a diagnosis of SMEI- Severe Myoclonic Epilepsy in Infancy) (25). According to the Franklin database, loss-of-function variants in this gene are pathogenic (26).

In a female patient (E100), aged 3 months, presenting with subcortical and periventricular tubers, hypomelanotic nevi, focal epilepsy, and diminished eye contact, we identified a novel variant in the *TSC2* gene (c.5068 + 2 T > C), Clinvar ID 165914071. This variant is absent in the Genome Aggregation Database (gnomAD) and segregates with tuberous sclerosis in the family. The patient’s father exhibits angiofibroma and normal intellectual development, and two brothers display multiple hypomelanotic nevi. The identified variant affects a donor splice site in intron 39 of the *TSC2* gene, and it is anticipated to disrupt RNA splicing. Disruption of this splice site has been observed in individual(s) with tuberous sclerosis complex (Invitae, unpublished data). In at least one individual the variant was observed to be *de novo*. It is splice-altering according to the Splice AI program (0.95, while score 1.00 is a maximal score, high probability of the variant being splice-altering) (27), dbscSNV Ada (0.98, while score 1.00 is a maximal score), dbscSNV RF (0.55, while 1 is the highest score) (28). In HGMD there are 117 pathogenic splicing variants in the *TSC2* gene.

Furthermore, in a female patient (E49) with developmental and epileptic encephalopathy characterized by seizures of various types, starting at 7 weeks of age and resistant to antiseizure medication, we identified a deletion of exons 4–18 in the *CDKL5* gene. The patient exhibits features reminiscent of Rett syndrome, including bruxism, hyperventilation, small hands, and feet. This deletion, found to be out of frame, introduces a premature translational stop signal, resulting in an absent or disrupted protein product. Notably, this variant was not detected in the patient’s parents. According to Franklin LOF variants in this gene are pathogenic (26). There are 48 gross deletions in this gene in HGMD, causing developmental and epileptic encephalopathy (29).

Additionally, another novel variant in the *CDKL5* gene, a frameshift variant [c.2009_2012dup, p.(Thr672Argfs*12), Clin Var ID 2026466], was identified in a 3-month-old girl (E131) presenting with recurrent myoclonic and focal seizures, oculogyric crises, and poor eye contact. This variant, absent in gnomAD, is expected to result in an absent or disrupted protein product. Parental samples were not tested for this variant.

In a 7-year-old girl (E55) with focal epilepsy and a normal brain MRI, we identified a novel *de novo* variant (c.629 + 1G > T, Clinvar ID 1489034) in the *NPRL3* gene. This variant, absent in gnomAD, affects a donor splice site in intron 7 of the *NPRL3* gene. Disruption of this splice site has been previously observed in individuals with clinical features consistent with NPRL3-related conditions (data unpublished by Invitae). According to Splice-AI it is splice-altering (score 0.97) (27). According to Franklin, LOF variants in this gene are pathogenic (26). Splicing variants in this gene have not been recorded in HGMD (29).

In a severely affected 13-month-old girl (E128) presenting with focal epilepsy since infancy, deafness, lack of eye contact, severe global developmental delay, and brain malformations including cortical heterotopia, pachygyria, and enlarged ventricles, a novel *de novo* variant in the *DYNC1H1* gene was identified: c.10016G > A, p.(Arg3339His). This variant is not present in the Genome Aggregation Database (gnomAD). Notably, it has been observed in individuals exhibiting clinical features consistent with DYNC1H1-related intellectual disability (data unpublished by Invitae). Biophysical properties and protein sequence modeling performed at Invitae suggest that this missense variant is likely to disrupt the function of the DYNC1H1 protein. According to many predictive tools the variant is disease-causing or probably damaging [SIFT score 0.03 (deleterious), Polyphen- score 1.0, damaging, Mutation Taster- disease-causing, Mutation Assessor- score 3.34, pathogenic (30–33)].

**Figure 4:**
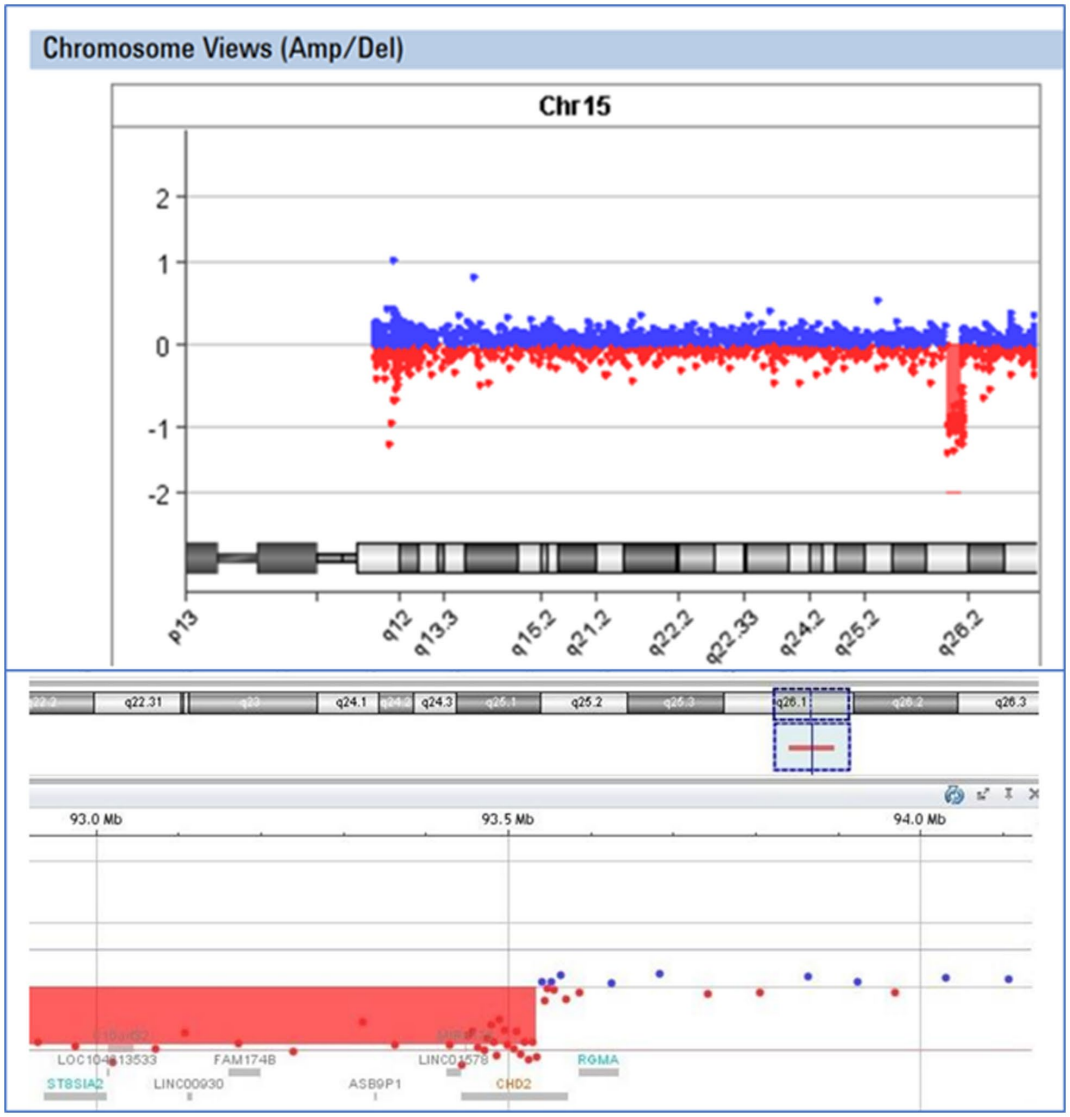
Visual presentation of a deletion 15q26.1, identified by array-CGH (Agilent SurePrint G3 CGH ISCA v2, 8x60K) in patient E43, with a deletion of the *CHD2* gene.

In the case of patient E43, an 8-year-old boy exhibiting epilepsy characterized by atonic seizures, focal seizures, and permanent EEG changes since the age of 16 months, along with intellectual disability and microcephaly, we identified a deletion encompassing exons 2–28 of the *CHD2* gene. Oligonucleotide array Comparative Genomic Hybridization (aCGH) analysis using the Agilent GenetiSure Cyto CGH Microarray 8x60K revealed an abnormal male pattern with a deletion in the long arm (q) of chromosome 15 (region 15q26.1; size of the detected deletion: 1.8 Mb; genomic position according to HG19: 91737109_93534368) (Figure 4). Loss-of-function mutations in the *CHD2* gene are known to be pathogenic (34).

Furthermore, in a 6-year-old girl manifesting a phenotype resembling atypical Rett syndrome (including bruxism, lack of speech, focal epilepsy since the age of 2 years, severe motor delay, stereotypies, and no hand apraxia), we identified a novel frameshift *de novo* variant in the DDX3X gene: c.1658_1661del, p.(Thr553Argfs*18). This variant is not present in the Genome Aggregation Database (gnomAD) and is expected to result in an absent or disrupted protein product. Loss of function variants in *DDXCX* gene are pathogenic, according to Franklin (26).

Supplementary Table S1 presents detailed clinical and molecular data of all 30 patients with the identified causative variant (novel and already described).

### Detection of other copy number variants with TNGSP

In three patients, the identification of larger chromosomal deletions was prompted by the suspicion that such deletions might be present, based on the observation of the deletion of an entire gene or its initial or final segment.

Patient E56 exhibited a common deletion in the 16p11.2 region, initially detected by the panel as a deletion encompassing the entire *PRRT2* gene and subsequently confirmed through Multiplex Ligation-dependent Probe Amplification (MLPA).

In the case of patient E7, a 43-year-old man and the eldest in the cohort, the presence of a deletion affecting the entire *UBE3A* and *GABRB3* genes led to the suspicion of Angelman syndrome. This diagnosis was subsequently confirmed by MLPA, where all three probes in the SALSA^®^ MLPA^®^ P245 Probemix specific to the 15q11.2 region indicated a heterozygous deletion. Despite lacking the typical behavioral features associated with Angelman syndrome, patient E7 exhibited dark hair, brown eyes, severe intellectual disability, absent speech, and early-onset epilepsy. Notably, the NGS panel was the initial diagnostic test conducted for this patient.

**Figure 5:**
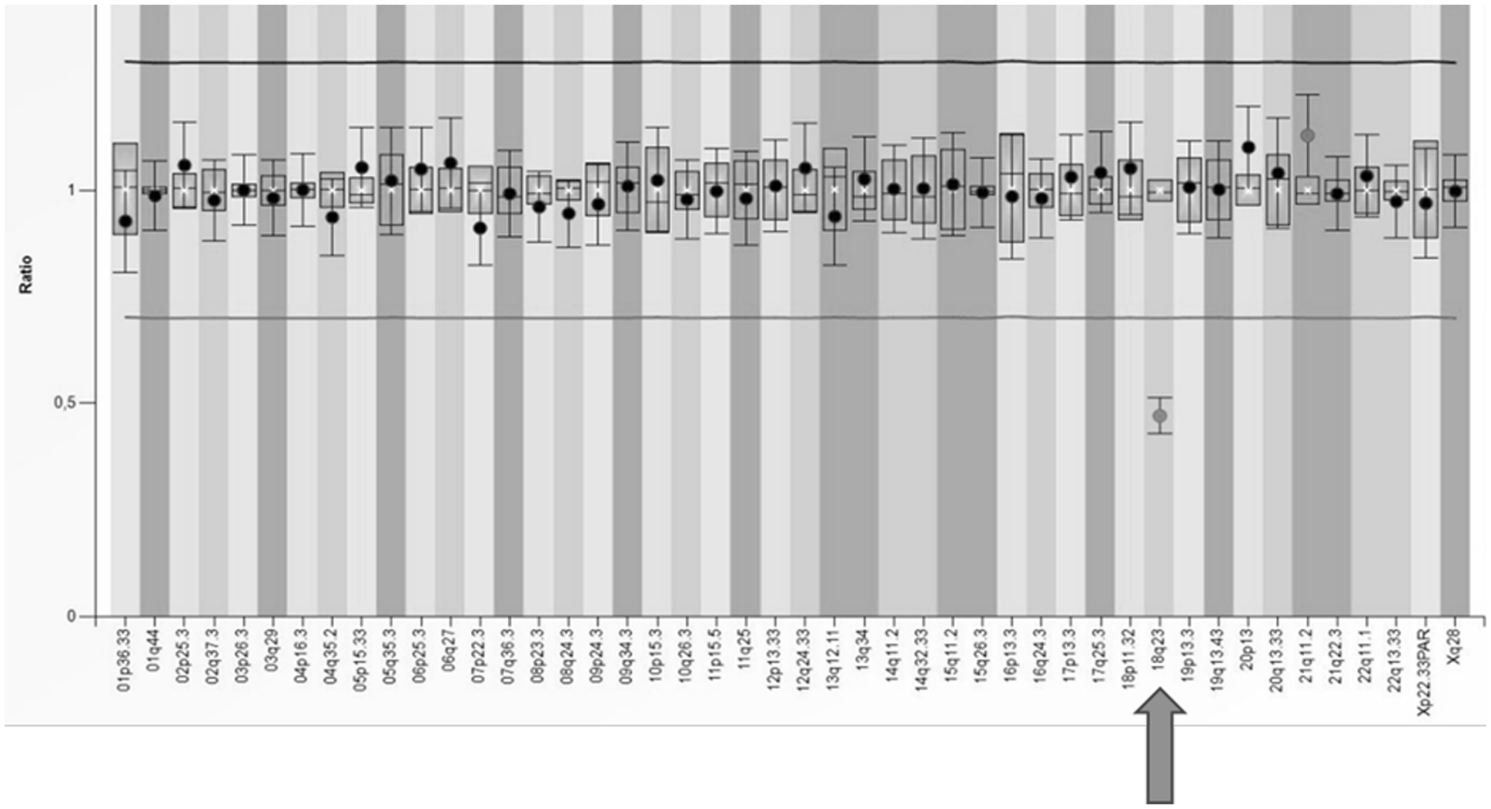
The results of MLPA analysis using SALSA^®^ MLPA^®^ P070 Probemix and Coffalyser Net software in a patient with a deletion in the subtelomeric region of the long arm of chromosome 18 (indicated by an arrow).

In our cohort, one patient received a dual diagnosis of epilepsy with both a documented alteration in the *TSC2* gene and a substantial deletion within the long arm of chromosome 18. This individual exhibited symptoms of tuberous sclerosis and focal epilepsy from early infancy. However, the patient also manifested a syndrome of multiple defects that could not be solely attributed to tuberous sclerosis, including microphthalmia and coloboma of the iris. Beyond the pathogenic variant identified in the *TSC2* gene through the NGS panel, a significant finding was the detection of a deletion encompassing the entire *RAX* gene associated with autosomal recessive microphthalmia. Confirmation of the heterozygous deletion in the 18q23 region was achieved through two MLPA tests (SALSA^®^ MLPA^®^ Probemix P036 and P070), specifically using the probe designed for this region (Figure 5). Subsequent array CGH analysis unveiled the presence of an 18q21.3q23 deletion, leading to the conclusive diagnosis of 18q deletion syndrome in this patient.

## Discussion

### The yield of TNGSP in patients with epilepsy

The yield of multigene tests based on next-generation sequencing in the studied group of Polish patients with epilepsy was 21.7%. It does not differ significantly from the diagnostic yield in other populations. Leduc-Passah et al. tested 227 patients with the TNGSP, achieving an efficiency of 17% (35). The most frequently impaired genes were similar to those identified in our cohort: *SCN1A, KCNQ2, PCDH19,* and *PRRT2* (35). In a Scottish study, among all consecutive children under 3 years of age with epilepsy, the yield was 24% (4). Symonds et al. found a diagnostic variant in one of nine genes in 66% of their patients (*PRRT2, SCN1A, KCNQ2, SLC2A1, TSC2, CDKL5, DEPDC5, PCDH19*, and *SLC6A2*). We identified a diagnostic variant in seven genes from this list, even though the ethnicity of the patients in our cohort was different. Costain et al. achieved an efficiency of 19.7%, Fernandez- 23% (5, 36). The larger the panel, the greater the diagnostic yield, especially in patients without DEE (8). In all patients, we used large panels of more than 100 genes.

### The yield of TNGSP in different endophenotypes of epilepsy

Willimsky et al. showed that the diagnostic yield significantly depended on patient age when epilepsy occurred (32% less than 1 year old, 1% more than 3 years old) (8). Meta-analysis of 72 research studies by Stefanski et al. showed that in the group of patients with epilepsy, the overall diagnostic yield of NGS techniques (panels, WES) was 29.3% in neonatal/infantile seizures (37). Our research shows that in children who started having seizures before the age of 13 months, it was nearly 6 times more likely to discover the cause of monogenic epilepsy. This is due only in part to the early start of seizures in DEE but also to the infantile manifestation of focal seizures in benign familial infantile epilepsies, which may be caused by variants in the *PRRT2, KCNQ2, SCN2A*, like in our cohort.

There were no statistically significant differences in the diagnostic yield of the test between the sexes.

The type of seizures influenced the yield in our cohort. It was the highest in DEE, like in other populations. It was surprisingly high in patients with focal seizures and lowest in patients with generalized seizures. It is difficult to find a similar relationship in the works of other authors. It was usually found that the monogenic cause of epilepsy is most often determined in patients with DEE, less often in epilepsy with generalized seizures, and less often in epilepsy with focal seizures (37). We reached statistical significance only for the differences between patients with epileptic encephalopathies and patients with generalized seizures (*p* = 0.01817).

In our group, there is a clear relationship between the occurrence of seizures in a child with elevated body temperature and the chance of detecting a genetic variant. A genetic variant was found in 25.9% of children with seizures sensitive to elevated temperature and in 10.7% of children with seizures not related to fever. Still, this relationship did not reach statistical significance. The association of seizures with temperature in the work of Bayat et al. increased the chance of detecting a genetic cause by WES and array-CGH (72% of “solved cases” had a history of febrile seizures) (16).

On the other hand, the presence of dysmorphic features and/or congenital malformations slightly lowered the chances of detecting the causative variant in our group (diagnostic yield of 10.7% in the group with dysmorphic features/MCA and 24.5% in the group without any dysmorphic features/MCA). This relationship may be coincidental, although, from a clinical point of view, patients with congenital malformations and/or significant facial dysmorphic features are first screened by clinical microarray in search of rare syndromes of chromosomal micro aberrations. In these patients we consider also rare dysmorphic syndromes, mostly not covered by TNGSP, better detectable in WES analysis. In the extensive work of Costain et al., the chance of detecting a variant by TNGSP and WES among patients with dysmorphic features was 31% (5). The same author recommends the TNGSP as a first-choice test in children with onset of seizures less than 3 years of age with no dysmorphic features and normal brain MRI (5).

In our study, all children with epilepsy underwent testing with TNGSP, irrespective of the presence or absence of abnormalities in MRI brain imaging. Indeed, it is noteworthy that some authors advocate for the exclusion of children with structural brain lesions from analyses. However, the impact of this exclusion on the yield of genetic testing appears to be inconsistent across studies. In Korean studies, even after implementing this exclusion criterion, the yield of genetic testing in patients with epileptic encephalopathies remained comparable to our cohort, reaching 37.8 and 37.1% with a panel of 172 genes (38). Lee analyzed patients with early-onset epilepsy lacking structural abnormalities on brain MRI, utilizing a panel of 76 genes, and achieved a high yield of 34.5% (17).

Costain et al. observed that, in their cohort, the presence of any abnormality in brain MRI did not significantly alter the yield of genetic testing. However, it’s noteworthy that their group seemingly lacked patients with cortical dysplasia (5). The likelihood of detecting a genetic variant increased slightly with the presence of white matter hyperintensities, corpus callosum abnormalities, and myelin abnormalities, while brain atrophy had a mild lowering effect on the diagnostic yield. Intriguingly, white matter hyperintensities (rather related to environmental injury) were identified in 32.4% of patients with a genetic diagnosis and in 24.2% of patients without any genetic diagnosis (5).

In the aforementioned study, a total of 72.1% of patients with a confirmed genetic diagnosis (following Targeted Next-Generation Sequencing Panel or Whole Exome Sequencing) exhibited brain MRI abnormalities. In contrast, 63.7% of patients without a discerned genetic diagnosis demonstrated such abnormalities (5).

MRI findings suggestive of an environmental cause of epilepsy contributed to a reduction in the diagnostic yield within our cohort, amounting to 18.8%, compared to the overall yield of 27.7% observed in the entire group of patients with available imaging data.

Among the 94 patients studied by us with available MRI data, 14 exhibited imaging abnormalities indicative of a genetic origin of epilepsy. These abnormalities encompass cortical dysplasia, characteristic cortical and subcortical alterations associated with tuberous sclerosis, hypoplasia of the cerebellum, and cerebral atrophy. Remarkably, in 7 of these 14 patients with such findings, we successfully identified a pathogenic or likely pathogenic (P/LP) variant in one of the genes examined. For the remaining 7 patients, there is merit in pursuing further genetic testing through Whole Exome Sequencing (WES) analysis and array-CGH analysis to explore potential small copy number variations (CNVs) and investigate exceedingly rare syndromes.

In general, patients without a clear diagnosis after TNGSP should undergo further analysis using either WES or WGS. Both WES and WGS are robust techniques that allow the analysis of all coding sequences in the genome. One additional positive aspect is the possibility of reanalysis, which can be valuable for updating information or identifying new findings over time. Reanalysis should be considered after 6–12 months (39). WGS permits the analysis of 5′UTR [5′ untranslated region) variants and small Copy Number Variations (CNVs)]. According to the study of Grether et al., when comparing the coverage of the coding region between WES with 120x coverage and 30x WGS, genome sequencing improved coverage in only 1% of the exome (6). Lionel et al. advocate for Whole Genome Sequencing (WGS) as the primary genetic test, particularly for individuals presenting with neurological, ophthalmological, and psychological disorders. Their study involved the examination of 103 patients exhibiting diverse neurological, ophthalmological, and neurodevelopmental conditions, with 8.7% of the subjects born from consanguineous unions.

The implementation of Whole Genome Sequencing identified a diagnostic variant in 41% of the pediatric cohort. Notably, in cases where both Whole Exome Sequencing (WES) and WGS were employed, 25.7% of the children exhibited variants that were exclusively detected by WGS. These encompassed deep intronic Single Nucleotide Variants (SNVs), small Copy Number Variations (CNVs), SNVs within non-coding RNA, mitochondrial variants, and exonic SNVs within regions inadequately covered by WES.

The findings of the study underscore the superior diagnostic efficacy of WGS compared to WES, emphasizing its capacity to uncover variants that may elude detection by the latter. This is particularly pertinent in instances involving consanguinity and autosomal recessive conditions (7).

### X-linked inheritance in females

Interestingly, pathogenic variants in genes located on the X chromosome were found in five girls. These were variants in the gene *CDKL5* (three girls), *NEXMIF* (one girl), and *DDX3X* (one girl) (Supplementary Table S1). No variants in the genes on the X chromosome were found in the males in the study group. The higher incidence of variants in genes located on the X chromosome in girls may come as a surprise. This result did not reach statistical significance; however, it may be an impulse to study this relationship in larger groups of patients. Variants in genes on the X chromosome are classically associated with symptoms of intellectual disability in boys, less often with a milder degree of intellectual disability in females (40, 41). However, it has long been known that in the case of epilepsy, the matter is much more complicated. There are genetic syndromes caused by variants in genes on the X chromosome that are restricted to females or occur significantly more often in females, such as epileptic and developmental encephalopathy associated with the *PCDH19* gene (42, 43). As males with a mosaic variant may be affected, cellular interference as the pathogenic mechanism is postulated. In the case of *SMC1A*, gene missense variants cause Cornelia de Lange (CDLs) in both males and females (44). Still, loss-of-function variants are found only in females without the phenotype of CDLs but presenting with epilepsy (45). Missense variants in *ALG13* are found mainly (but not only) in females with severe infantile spasms (46). Finally, there are genes, such as *IQSEC2*, *CDKL5,* and *NEXMIF*, whose alteration causes epilepsy in both sexes. In the work of Bayat et al., variants in genes related to the X chromosome were found in ten cases (15). Five of the six genes found to be damaged may cause epilepsy in females (*PCDH19, MECP2, IQSEC2, CDKL5, NEXMIF*) (15). Lindy et al. studied 8,565 patients with epilepsy with a panel of 70 genes and array-CGH. In 7.6% of positive cases in the cohort, a causative variant was found in the *CDKL5* gene; in 5.7% a causative variant in *PCDH19*; in 3.5% in *MECP2* (3). McKnight et al. first noted a significantly higher diagnostic yield of testing with TNGSP in female individuals with epilepsy and intellectual disability/developmental delays than in male individuals with epilepsy plus intellectual disability/developmental delays (2). According to these authors, it is mainly due to diagnostic findings in X-linked genes. The idea of the predominance of males in X-linked epilepsy should be revised. It may turn out that in large groups of patients with epilepsy and variants in genes on the X chromosome, girls predominate.

### CNVs detected in the cohort under study

The cases with CNVs detected in the frame of this study underscore the importance of vigilance in cases where the initial test is a Targeted Next-Generation Sequencing Panel (TSNGSP). Special attention should be given to heterozygous changes involving the complete deletion of a gene or a substantial fragment. If such changes occur in a gene associated with an autosomal recessive disorder, there is a risk of overlooking their significance. Consequently, it becomes imperative to explore the possibility of larger chromosomal alterations that may encompass additional genes not covered by the panel, requiring the use of MLPA or array-CGH for comprehensive assessment.

### Benefits of determining the cause of epilepsy

The identification of a monogenic cause of epilepsy confers several potential benefits, primarily revolving around the prospect of modifying antiepileptic treatment strategies. Notably, variants in frequently implicated genes such as *SCN1A*, *TSC2*, *PRRT2*, and *SCN2A* may prompt alterations in clinical management, including the introduction of specific antiepileptic drugs or the avoidance of certain medications. While this study did not delve into the introduction of these modifications and their efficacy, insights from studies by Belestrini et al. offer a cautionary perspective. In their cohort of 84 children with recognized monogenic causes of epilepsy, personalized therapy was feasible for only 56 individuals, with implementation in 33 children. The reduction in seizure frequency by more than 50% was achieved in merely 10 children (47).

Bayat et al. contributed valuable observations on personalized seizure treatment in patients with monogenic epilepsy causes. Although the study group, drawn from The Danish Epilepsy Center, a tertiary hospital in Denmark, likely influenced the results (encompassing a substantial number of patients with refractory epilepsy), precision therapy following genetic testing demonstrated effectiveness in 93% of patients. However, only 12.5% achieved seizure freedom (16).

For specific genetic variants, such as those in *SLC2A1*, the ketogenic diet has shown remarkable efficacy in substantially reducing seizure frequency. The prognosis for children with variants in *PRRT2* is generally favorable for further development and a positive response to antiepileptic drugs. Variants in genes like *DEPDC5*, *NPRL3*, and others within the GATOR1 pathway emphasize the imperative to conduct imaging tests for the localization of epileptic foci. Genetic counseling is always possible when a pathogenic variant is identified. It is difficult for genes with reduced penetrance (*DEPDC5, SCN1A, KCNQ2*), various expressivity, and located on the X chromosome. In the latter case, it is challenging due to the possibility of a lack of manifestation in female or male relatives.

### Limitations

The main limitation of this study is that it is a retrospective analysis of patients who underwent TNGSP testing at their own expense. In Poland, patients with epilepsy are referred to a genetic clinic by their family doctor, pediatrician, or child neurology specialist. Not all patients are referred to a genetic clinic, and their referral does or does not depend on the referring physician’s experience, determination, and knowledge. It can be assumed that patients with treatment-resistant seizures, dysmorphic features, congenital developmental defects, or developmental delays are more often referred to a genetic clinic. Polish patients can also benefit from consultations without a referral from a family doctor or a pediatric neurologist. They are driven by the desire to determine the cause of epilepsy because they plan to have another child. This may result in the younger age of patients being tested. Not all patients have the opportunity to reach a genetic clinic located in a large academic center. The test cost, which is not refundable, is also an obstacle for many Polish families. For the above reasons, the study group is not representative of the entire Polish group of epilepsy patients.

The decision to use either saliva or blood as the source of DNA for Next-Generation Sequencing (NGS) in this study was made on an individual basis, taking into account parental preferences and the availability of a pediatric nurse. Both blood and saliva are commonly employed for DNA extraction in NGS studies. A study published in BMC Medical Genomics compared the quality of whole genome sequencing (WGS) between blood and saliva-derived DNA in cardiac patients. The findings indicated that high-quality saliva samples meeting stringent quality control criteria can be effectively utilized for WGS, especially when blood-derived DNA is either unavailable or deemed unsuitable (48).

The study noted that saliva-derived DNA provides a satisfactory yield of Single Nucleotide Variant (SNV) calls but exhibits a lower yield for Copy Number Variant (CNV) calls in comparison to blood-derived DNA (48). Another study that compared genetic variant detection from human whole blood, saliva, and buccal samples found that the quality of DNA obtained from saliva and buccal samples is comparable to that from blood samples (49). Notably, the choice of DNA source may introduce biases into the analyses, as exemplified by a study demonstrating the presence of a variant in a mosaic state in the *SMC1A* gene in a patient with Cornelia de Lange syndrome, which was detectable in DNA extracted from saliva but not in blood-derived DNA (50). Therefore, the selection of DNA sources warrants careful consideration to mitigate potential biases in the analytical outcomes.

The utilization of panels with varying gene counts represents a limitation of this study, as panels with a greater number of genes may potentially offer enhanced diagnostic efficiency. Truty et al. studied the yield of TNGSP in a large group of 9,769 patients with epilepsy (21). Eight genes constituted 50% of positive diagnoses, 22 genes contributed to 80%, whereas the remaining 76 genes made a 20% contribution to positive diagnoses (21). It proves that even smaller panels than the ones used in our study should prove adequate in elucidating a significant proportion of cases, provided they include the key players in the genetics of epilepsy. The variance in the number of genes analyzed in different patients within the study cohort is attributed to several factors. Primarily, the increasing number of genes tested by the Invitae laboratory over subsequent years contributes significantly to the observed differences. Additionally, the personal choices of the consulting clinical geneticist played a role in determining the scope of genetic testing for individual patients. For instance, broader testing was opted for in more complex cases, while a lower number of genes were analyzed in instances where there was a suspicion of a specific syndrome. Notably, the smallest panels, applied in only two individuals with suspected tuberous sclerosis (comprising 104 and 150 genes), lacked coverage for the *PRRT2* gene. In all other cases, a comprehensive panel encompassing all genes associated with identified pathogenic and potentially pathogenic variants was employed. This variability in the gene panel size underscores the evolving landscape of genetic testing capabilities and the importance of tailored approaches based on the clinical context and suspected genetic etiology.

In several cases, familial variant testing could not be performed. This is due to the lack of parental consent to such tests. Refusal to undergo a family test may be due to various reasons: fear of revealing the person as an asymptomatic carrier of a variant in the case of an incompletely penetrant or X-linked gene (which may make the carrier feel guilty), not planning to have more children and lack of interest in family tests for this reason, perhaps also for other, more complicated reasons (fear of revealing incorrectly assigned paternity). In the case of potentially pathogenic variants, determining whether the variant arose *de novo* or was inherited from a healthy parent is crucial. Parents’ decisions must be respected and cannot be questioned, even in such a case.

## Conclusion

Research utilizing panels based on next-generation sequencing (NGS) in the Polish population has revealed a monogenic cause of epilepsy in over 20% of patients. These identified variants are found in genes whose defects have been previously identified in both European and non-European populations, indicating a common etiology of epilepsy in different parts of the world. Notably, a meticulous analysis of heterozygous variants in the form of gene copy number changes has the potential to unveil more extensive chromosomal aberrations.

An intriguing observation is the relatively frequent occurrence of pathological variants in genes associated with the X chromosome in girls. This pattern warrants further in-depth research to elucidate the underlying mechanisms and implications of such occurrences in the context of epilepsy.

## Data availability statement

The datasets presented in this study can be found in online repositories. The names of the repository/repositories and accession number(s) can be found in the article/Supplementary material.

## Ethics statement

The requirement of ethical approval was waived by Ethical Committee of Poznan University of Medical Sciences for the studies involving humans because according to Polish law, no ethical approval is needed for retrospective clinical analyses. The studies were conducted in accordance with the local legislation and institutional requirements. Written informed consent for participation in this study was provided by the participants’ legal guardians/next of kin.

## Author contributions

MB-S: Conceptualization, Data curation, Investigation, Methodology, Software, Supervision, Visualization, Writing – original draft, Writing – review & editing. KW: Data curation, Investigation, Validation, Writing – review & editing. AW-W: Data curation, Investigation, Methodology, Conceptualization, Writing – review & editing. JM: Conceptualization, Investigation, Methodology, Software, Data curation, Writing – review & editing. DK: Data curation, Investigation, Writing – review & editing. DT-K: Data curation, Investigation, Writing – review & editing. MiP: Data curation, Investigation, Methodology, Validation, Writing – review & editing. MaP: Data curation, Investigation, Validation, Writing – review & editing. NK: Data curation, Investigation, Validation, Writing – review & editing. BS: Data curation, Investigation, Writing – review & editing.

## References

[1] Ottman R Hirose S Jain S Lerche H Lopes-Cendes I Noebels JL. Genetic testing in the epilepsies - Report of the ILAE Genetics Commission. Epilepsia. (2010). 51. doi: 10.1111/j.1528-1167.2009.02429.x PMC2855784 20100225

[2] McKnight D Bristow SL Truty RM Morales A Stetler M Westbrook MJ. Multigene panel testing in a large cohort of adults with epilepsy: diagnostic yield and clinically actionable genetic findings. Neurology. (2022) 8:e650. doi: 10.1212/NXG.0000000000000650, PMID: 34926809 PMC8678910

[3] Lindy AS Stosser MB Butler E Downtain-Pickersgill C Shanmugham A Retterer K. Diagnostic outcomes for genetic testing of 70 genes in 8565 patients with epilepsy and neurodevelopmental disorders. Epilepsia. (2018) 59:1062–1071. doi: 10.1111/epi.14074 29655203

[4] Symonds JD Elliott KS Shetty J Armstrong M Brunklaus A Cutcutache I. Early childhood epilepsies: epidemiology, classification, aetiology, and socio-economic determinants. Brain. (2021) 144:2879–2891. doi: 10.1093/brain/awab162 34687210 PMC8557326

[5] Costain G Cordeiro D Matviychuk D Mercimek-Andrews S. Clinical application of targeted next-generation sequencing panels and whole exome sequencing in childhood epilepsy. Neuroscience. (2019) 418:291–310. doi: 10.1016/j.neuroscience.2019.08.016, PMID: 31487502

[6] Grether A Ivanovski I Russo M Begemann A Steindl K Abela L. The current benefit of genome sequencing compared to exome sequencing in patients with developmental or epileptic encephalopathies. Mol Genet Genom Med. (2023) 11:e2148. doi: 10.1002/mgg3.2148, PMID: 36785910 PMC10178799

[7] Lionel AC Costain G Monfared N Walker S Reuter MS Hosseini SM. Improved diagnostic yield compared with targeted gene sequencing panels suggests a role for whole-genome sequencing as a first-tier genetic test. Genet Med. (2018) 20:435–43. doi: 10.1038/gim.2017.119, PMID: 28771251 PMC5895460

[8] Willimsky EK Munzig A Mayer K Biskup S Abicht A Hoertnagel K. Next Generation Sequencing in Pediatric Epilepsy Using Customized Panels: Size Matters. Neuropediatrics. (2021) 52:92–97. doi: 10.1055/s-0040-1712488 33086385

[9] Hirsch E French J Scheffer IE Bogacz A Alsaadi T Sperling MR. ILAE definition of the idiopathic generalized epilepsy syndromes: position statement by the ILAE task force on nosology and definitions. Epilepsia. (2022) 63:1475–99. doi: 10.1111/epi.17236, PMID: 35503716

[10] Berkovic SF Mulley JC Scheffer IE Petrou S. Human epilepsies: interaction of genetic and acquired factors. Trends Neurosci. (2006) 29:391–7. doi: 10.1016/j.tins.2006.05.009 16769131

[11] Maloney EM Corcoran P Costello DJ O’Reilly ÉJ. Association between social deprivation and incidence of first seizures and epilepsy: A prospective population-based cohort. Epilepsia. (2022) 63: 2108–19. doi: 10.1111/epi.17313 35611982 PMC9544186

[12] Baldassari S Picard F Verbeek NE van Kempen M Brilstra EH Lesca G. The landscape of epilepsy-related GATOR1 variants. Genet Med. (2019) 21:398–408. doi: 10.1038/s41436-018-0060-2, PMID: 30093711 PMC6292495

[13] Shen K Huang RK Brignole EJ Condon KJ Valenstein ML Chantranupong L. Architecture of the human GATOR1 and GATOR1-rag GTPases complexes. Nature. (2018) 556:64–9. doi: 10.1038/nature26158, PMID: 29590090 PMC5975964

[14] McKnight D Morales A Hatchell KE Bristow SL Bonkowsky JL Perry MS. Genetic testing to inform epilepsy treatment management from an international study of clinical practice. JAMA Neurol. (2022) 79:1267–76. doi: 10.1001/jamaneurol.2022.3651, PMID: 36315135 PMC9623482

[15] Bayat A Bayat M Rubboli G Møller RS. Epilepsy syndromes in the first year of life and usefulness of genetic testing for precision therapy. Genes (Basel). (2021) 12:1051. doi: 10.3390/genes12071051 34356067 PMC8307222

[16] Bayat A Fenger CD Techlo TR Højte AF Nørgaard I Hansen TF. Impact of Genetic Testing on Therapeutic Decision-Making in Childhood-Onset Epilepsies—a Study in a Tertiary Epilepsy Center. Neurotherapeutics, (2022) 19. doi: 10.1007/s13311-022-01264-1 PMC9587146 35723786

[17] Lee J Lee C Ki CS Lee J. Determining the best candidates for next-generation sequencing-based gene panel for evaluation of early-onset epilepsy. Mol Genet Genom Med. (2020) 8:e1376. doi: 10.1002/mgg3.1376, PMID: 32613771 PMC7507365

[18] World Medical Association. World Medical Association declaration of Helsinki: Ethical principles for medical research involving human subjects. JAMA. (2013) 310:2191–4. doi: 10.1001/jama.2013.281053 24141714

[19] Scheffer IE Berkovic S Capovilla G Connolly MB French J Guilhoto L. ILAE classification of the epilepsies: position paper of the ILAE Commission for Classification and Terminology. Epilepsia. (2017) 58:512–21. doi: 10.1111/epi.13709, PMID: 28276062 PMC5386840

[20] American Psychiatric Association (APA). Diagnostic and Statistical Manual of Mental Disorders: Neurodevelopmental Disorders. Diagnostic and Statistical Manual of Mental Disorders. (2013).

[21] Truty R Patil N Sankar R Sullivan J Millichap J Carvill G. Possible precision medicine implications from genetic testing using combined detection of sequence and intragenic copy number variants in a large cohort with childhood epilepsy. Epilepsia Open. (2019) 4:397–408. doi: 10.1002/epi4.12348, PMID: 31440721 PMC6698688

[22] Lincoln SE Truty R Lin CF Zook JM Paul J Ramey VH. A rigorous Interlaboratory examination of the need to confirm next-generation sequencing-detected variants with an orthogonal method in clinical genetic testing. J Mol Diagn. (2019) 21:318–29. doi: 10.1016/j.jmoldx.2018.10.009, PMID: 30610921 PMC6629256

[23] Truty R Paul J Kennemer M Lincoln SE Olivares E Nussbaum RL. Prevalence and properties of intragenic copy-number variation in Mendelian disease genes. Genet Med. (2019) 21:114–23. doi: 10.1038/s41436-018-0033-5, PMID: 29895855 PMC6752305

[24] Nykamp K Anderson M Powers M Garcia J Herrera B Ho YY. Sherloc: A comprehensive refinement of the ACMG-AMP variant classification criteria. Genet Med. (2017) 19:1105–17. doi: 10.1038/gim.2017.37 28492532 PMC5632818

[25] Fukuma G Oguni H Shirasaka Y Watanabe K Miyajima T Yasumoto S. Mutations of neuronal voltage-gated Na+ channel α1 subunit gene SCN1A in Core severe myoclonic epilepsy in infancy (SMEI) and in borderline SMEI (SMEB). Epilepsia. (2004) 45:140–8. doi: 10.1111/j.0013-9580.2004.15103.x, PMID: 14738421

[26] Available at: https://franklin.genoox.com - Franklin by Genoox (n.d.).

[27] Jaganathan K Kyriazopoulou Panagiotopoulou S McRae JF Darbandi SF Knowles D Li YI. Predicting splicing from primary sequence with deep learning. Cells. (2019) 176:535–548.e24. doi: 10.1016/j.cell.2018.12.015 30661751

[28] Jian X Boerwinkle E Liu X. In silico prediction of splice-altering single nucleotide variants in the human genome. Nucleic Acids Res. (2014) 42:13534–44. doi: 10.1093/nar/gku1206, PMID: 25416802 PMC4267638

[29] Stenson PD Mort M Ball EV Shaw K Phillips AD Cooper DN. The human gene mutation database: building a comprehensive mutation repository for clinical and molecular genetics, diagnostic testing and personalized genomic medicine. Hum Genet. (2014) 133:1–9. doi: 10.1007/s00439-013-1358-4, PMID: 24077912 PMC3898141

[30] Available at: http://genetics.bwh.harvard.edu/pph2/ (n.d.).

[31] Available at: http://mutationassessor.org/r3/ (n.d.).

[32] Available at: https://sift.bii.a-star.edu.sg/index.html (n.d.).

[33] Available at: https://www.mutationtaster.org/) (n.d.).

[34] Carvill GL Heavin SB Yendle SC McMahon JM O’Roak BJ Cook J. Targeted resequencing in epileptic encephalopathies identifies de novo mutations in CHD2 and SYNGAP1. Nature Genetics. (2013) 45:825–30. doi: 10.1038/ng.2646 23708187 PMC3704157

[35] Leduc-Pessah H White-Brown A Hartley T Pohl D Dyment DA. The Benefit of Multigene Panel Testing for the Diagnosis and Management of the Genetic Epilepsies. Genes. (2022) 13:872. doi: 10.3390/genes13050872 35627257 PMC9141259

[36] Fernández IS Loddenkemper T Gaínza-Lein M Sheidley BR Poduri A. Diagnostic yield of genetic tests in epilepsy: A meta-analysis and cost-effectiveness study. Neurology. (2019) 92:e418–e428. doi: 10.1212/WNL.0000000000006850 30610098 PMC6369901

[37] Stefanski A Calle-López Y Leu C Pérez-Palma E Pestana-Knight E Lal D. Clinical sequencing yield in epilepsy, autism spectrum disorder, and intellectual disability: A systematic review and meta-analysis. Epilepsia. (2021) 62. doi: 10.1111/epi.16755 PMC7839709 33200402

[38] Ko A Youn SE Kim SH Lee JS Kim S Choi JR. Targeted gene panel and genotype-phenotype correlation in children with developmental and epileptic encephalopathy. Epilepsy Res. (2018) 141:48–55. doi: 10.1016/j.eplepsyres.2018.02.003, PMID: 29455050

[39] Møller RS Larsen LHG Johannesen KM Talvik I Talvik T Vaher U. Gene panel testing in epileptic encephalopathies and familial epilepsies. Mol Syndromol. (2016) 7:210–9. doi: 10.1159/000448369, PMID: 27781031 PMC5073625

[40] Lehrke R. Theory of X-linkage of major intellectual traits. Am J Ment Defic. (1972) 76:626–31. PMID: 5031083

[41] Lubs HA Stevenson RE Schwartz CE. Fragile X and X-linked intellectual disability: four decades of discovery. In. Am J Hum Genet. (2012) 90:579–90. doi: 10.1016/j.ajhg.2012.02.018, PMID: 22482801 PMC3322227

[42] Depienne C Leguern E. PCDH19-related infantile epileptic encephalopathy: An unusual Xlinked inheritance disorder. Human Mutation, (2012) 33. doi: 10.1002/humu.22029 22267240

[43] Dibbens LM Tarpey PS Hynes K Bayly MA Scheffer IE Smith R. X-linked protocadherin 19 mutations cause female-limited epilepsy and cognitive impairment. Nature Genetics, (2008) 40. doi: 10.1038/ng.149 PMC2756413 18469813

[44] Deardorff MA Kaur M Yaeger D Rampuria A Korolev S Pie J. Mutations in cohesin complex members SMC3 and SMC1A cause a mild variant of Cornelia de Lange syndrome with predominant mental retardation. Am J Hum Genet. (2007) 80:485–94. doi: 10.1086/511888, PMID: 17273969 PMC1821101

[45] Jansen S Kleefstra T Willemsen MH de Vries P Pfundt R Hehir-Kwa JY. De novo loss-of-function mutations in X-linked SMC1A cause severe ID and therapy-resistant epilepsy in females: expanding the phenotypic spectrum. Clin Genet. (2016) 90:413–19. doi: 10.1111/cge.12729 26752331

[46] Ng BG Eklund EA Shiryaev SA Dong YY Abbott MA Asteggiano C. Predominant and novel de novo variants in 29 individuals with ALG13 deficiency: Clinical description, biomarker status, biochemical analysis, and treatment suggestions. J Inherit Metab Dis. (2020) 43. doi: 10.1002/jimd.12290 PMC7722193 32681751

[47] Balestrini S Chiarello D Gogou M Silvennoinen K Puvirajasinghe C Jones WD. Real-life survey of pitfalls and successes of precision medicine in genetic epilepsies. J Neurol Neurosurg Psychiatry. (2021) 92. doi: 10.1136/jnnp-2020-325932 PMC8458055 33903184

[48] Yao RA Akinrinade O Chaix M Mital S. Quality of whole genome sequencing from blood versus saliva derived DNA in cardiac patients. BMC Med Genet. (2020) 13:11. doi: 10.1186/s12920-020-0664-7, PMID: 31996208 PMC6988365

[49] Trost B Walker S Haider SA Sung WWL Pereira S Phillips CL. Impact of DNA source on genetic variant detection from human whole-genome sequencing data. J Med Genet. (2019) 56:809–17. doi: 10.1136/jmedgenet-2019-106281, PMID: 31515274 PMC6929712

[50] Garcia AG Malone J Li H. A novel mosaic variant on SMC1A reported in buccal mucosa cells, albeit not in blood, of a patient with Cornelia de Lange–like presentation. Cold Spring Harbor Mol Case Stud. (2020) 6:a005322. doi: 10.1101/MCS.A005322, PMID: 32532882 PMC7304356

