# Supplementary material for: Validation of targeted next-generation sequencing panels in a cohort of Polish patients with epilepsy: assessing variable performance across clinical endophenotypes and uncovering novel genetic variants
Source: Front Neurol. 2024 Jan 12;14:1316933. doi: 10.3389/fneur.2023.1316933 (PMC10849089; doi:10.3389/fneur.2023.1316933)
Supplement: Supplementary file 1 [file Table_1.DOCX]

| ID | Sex/ Age at examination | Clinical description | Relevant family history | Gene | Variant/type/inheritance/de novo or inherited | Description of the variant |
| --- | --- | --- | --- | --- | --- | --- |
| E10 | M/13.5 y | Focal epilepsy, first seizures at the age of 11 y, with laughter preceding loss of consciousness. Later, focal seizures with turning of the head to the right. Normal intellect. No cortical dysplasia in MRI of the brain. | Focal epilepsy in his sister, and mother healthy | *DEPDC5* | c.3259C>T, p.(Arg1087*)/het/ AD/ maternal inheritance, the same variant in his affected sister | ExAC- absent  Segregated with ADNFLE in a family(Picard et al., 2014). Experimental studies have shown that this nonsense change is targeted through nonsense-mediated mRNA decay, leading to a decrease or abolition of the level of mutated *DEPDC5* transcript (Picard et al., 2014)  ClinVar accession number SCV000814070.5 |
| E122 | M/7.5 y | Focal epilepsy, started at the age of 4 mo, drug-resistant. No dysmorphic features, normal MRI. Normal intellect, mild school problems, ADHD. |  | *DEPDC5* | Deletion (Entire coding sequence)/het/AD/NDA | A gross deletion of the genomic region encompassing the full coding sequence of the *DEPDC5* gene. The boundaries of this event are unknown as they extend beyond the assayed region for this gene and therefore may encompass additional genes. This variant has not been reported in the literature in individuals affected with DEPDC5-related conditions. |
| E41 | F/29 mo | First generalized tonic-clonic seizures at the age of 12 mo during infection, second after vaccination at 21 mo, followed by two tonic-clonic seizures without fever, interictal EEG normal. Normal development. Normal MRI of the brain. |  | *SCN1A* | c.4786C>T, p.(Arg1596Cys)/het/AD/NDA | The arginine 1596 residue is highly conserved. This variant is present in population databases (rs121917993, ExAC 0.002%). It has been observed to be de novo in several individuals with severe epilepsy(Dlugos et al., 2007; Harkin et al., 2007; Hoffman-Zacharska et al., 2015).  Algorithms developed to predict the effect of missense changes on protein structure and function (SIFT, PolyPhen-2, Align-GVGD) all suggest that this variant is likely to be disruptive. Other variant(s) that disrupt this residue have been observed in affected individuals (Zuberi et al., 2011). ClinVar accession number: SCV000933973.3 |
| E57 | F/14.5 y | Seizures since the age of 6 mo, always during infection, until the age of 14 y. Normal cognitive development. | Brother- seizures during infections, mother epilepsy until the age of 10 years. | *SCN1A* | c.811G>A, p.(Gly271Ser)/het/AD/maternal, affected brother with the same variant | GnomAD no frequency.  Has been observed in individual(s) with SCN1A-related conditions (Hız-Kurul et al., 2017)This variant disrupts the p.Gly271 amino acid residue in SCN1A. Other variant(s) that disrupt this residue have been determined to be pathogenic (Invitae). ClinVar accession number SCV002189251.2 |
| E73 | M/10 mo | Seizures during infection since the age of 4 mo, myoclonic, tonic-clonic, atonic. Epilepsy drug-resistant (on clobazam, stiripentol, walproate. |  | *SCN1A* | c.305T>C, p.(Phe102Ser)/AD/de novo | GnomAD no frequency. This missense change has been observed in individual(s) with clinical features of *SCN1A*-related conditions (Invitae). Advanced modeling of protein sequence and biophysical properties (such as structural, functional, and spatial information, amino acid conservation, physicochemical variation, residue mobility, and thermodynamic stability) performed at Invitae indicates that this missense variant is expected to disrupt SCN1A protein function. ClinVar accession number SCV002240638.2 |
| E34 | M/9 y | Focal seizures with secondary generalization since the age of 7 mo. Seizures are fever-induced. Microcephaly, mild dysmorphic signs. |  | *SCN1A* | c.5379del,  (p.Glu1794Lysfs*7)/AD/NDA | GnomAD no frequency. This sequence change creates a premature translational stop signal in the *SCN1A* gene. While this is not anticipated to result in nonsense-mediated decay, it is expected to disrupt the last 216 amino acid(s) of the SCN1A protein. This variant has not been reported in the literature in individuals affected with *SCN1A*-related conditions. This variant disrupts a region of the SCN1A protein in which another variant (p.Arg1912*) has been determined to be pathogenic (Fukuma et al., 2004) ClinVar accession number SCV002139937.2 |
| E98 | M/8 mo | Since the age of 7 mo myoclonic seizures, provoked by temperature, fever, bath. Mild hypotonia. |  | *SCN1A* | c.2836C>T,  p.(Arg946Cys)/AD/NDA | GnomAD no frequency.  This missense change has been observed in individual(s) with Dravet syndrome (PMID: Fukuma, Experimental studies have shown that this missense change affects SCN1A function (Volkers et al., 2011). This variant disrupts the p.Arg946 amino acid residue in SCN1A. Other variant(s) that disrupt this residue have been determined to be pathogenic (Verbeek et al., 2011). ClinVar accession number SCV000757715.4 |
| E56 | F/8 mo | Since the age of 4 mo episodes of focal seizures, in clusters. Initially the good reaction to CARB, then the reoccurrence of seizures, VAL was introduced with good effect (seizure-free). |  | *PRRT2* | Deletion (Entire coding sequence) confirmed by MLPA : microdeletion 16p11.2/het/AD/de novo | A gross deletion of the genomic region encompassing the full coding sequence of the *PRRT2* gene This particular deletion has been reported in the literature in individuals affected with epilepsy (Dale et al., 2012). |
| E127 | F/7 mo | Since the age of 5 mo focal seizures, recurrent, Hypotonia, mild developmental delay. No dysmorphic features. Normal build. |  | *PRRT2* | c.649dup, p.(Arg217Profs*8)/het/AD/de novo | This premature translational stop signal has been observed in individuals with infantile seizures (Chen et al., 2011). It has also been observed to segregate with disease in related individuals. ClinVar accession number SCV000291481.10 |
| E95 | M/17 mo | Focal epilepsy with prolonged seizures since the age of 7 mo. Poor reaction to LEV, good reaction to TOPI. Photosensitivity. Normal motor development, normal speech. Fast growth, quick dentition. | Maternal mother: one episode of fever-related seizures in infancy, migraine headaches, and her brother- cerebellar syndrome in adulthood. | *PRRT2* | c.649dup, (p.Arg217Profs*8)/het/AD/maternal |  |
| E1 | F/12 mo | Seizures since the age of 8 m, in clusters, without interictal EEG changes, no psychomotor retardation. Good reaction to VAL (absent seizures). | benign seizures of infancy in the mother and maternal brother. | *PRRT2* | c.649dup, (p.Arg217Profs*8)/het/AD/maternal |  |
| E84 | F/8 mo | Features of tuberous sclerosis: congenital heart rhabdomyomata, multiple subcortical tubers, periventricular tubers, SEGA tumor, focal epilepsy treated with vigabatrine, everolimus. Atypical features: coloboma of irises, lateral displacement of papillae, deafness, palatopharyngeal insufficiency, dolichocephaly, epicanthus, hypertelorism, anteverted nostrils, full upper lip, hemangiomas. |  | *TSC2* | c.880G>A, (p.Gly294Arg)/het/AD/de novo  In array-CGH a large deletion 18q21.3q23 de novo | GnomAD: no frequency. Observed in individual(s) with clinical features of tuberous sclerosis(Li et al., 2020; Strizheva et al., 2001). Advanced modeling of protein sequence and biophysical properties performed at Invitae indicates that this missense variant is expected to disrupt TSC2 protein function. ClinVar accession number: SCV003443036.1 |
| E100 | F/3 mo | Subcortical tubers, periventricular tubers, hypomelanotic nevi, focal epilepsy, poor eye contact. | Father mildly affected (angiofibromas, normal development), two brothers with the variant and multiple hypomelanotic nevi | *TSC2* | c.5068+2T>C/AD/paternal | Affects a donor splice site in intron 39 of the *TSC2* gene. It is expected to disrupt RNA splicing. GnomAD no frequency. Disruption of this splice site has been observed in individual(s) with tuberous sclerosis complex (Invitae). In at least one individual the variant was observed to be de novo.  ClinVar accession number: SCV000833744.3 |
| E138 | F/5 mo | Focal epilepsy, since the age of 1 mo, in the beginning, good reaction to VIGA, then a recurrence of seizures, now infantile spasms, developmental regression- poor eye contact, weak head control, axial hypotonia |  | *CDKL5* | c.532C>T  (p.Arg178Trp)/het/XL/maternal | GnomAD no frequency.  This missense change has been observed in individual(s) with early infantile epileptic encephalopathy (Artuso et al., 2010). This variant disrupts the p.Arg178 amino acid residue in CDKL5. Other variant(s) that disrupt this residue have been determined to be pathogenic(Nemos et al., 2009). ClinVar accession number SCV000639479.3 |
| E49 | F/8.5 y | Seizures started at the age of 7 weeks, drug-resistant epilepsy; different types of seizures: tonic, atonic, tonic-clonic, in series, developmental regression at the age of 3 y (lost ability to speak single words), at present profound intellectual disability. Some features of Rett syndrome: bruxism, hyperventilation, small hands and feet, no microcephaly. |  | *CDKL5* | Deletion (Exons 4-18)/het/XL/de novo | This variant is a gross deletion of the genomic region encompassing exon(s) 4-18 of the *CDKL5* gene. This deletion is out-of-frame, and is expected to create a premature translational stop signal and result in an absent or disrupted protein product. |
| E131 | F/3 mo | Since the age of 1 month myoclonic and focal seizures, recurrent, with poor reaction to AED. Hypotonia. Oculogyric crises. Poor eye contact. |  | *CDKL5* | c.2009_2012dup  (p.Thr672Argfs*12)/het/NDA | It is expected to result in an absent or disrupted protein product. Loss-of-function variants in CDKL5 are known to be pathogenic (Fehr et al., 2013). GnomAD no frequency. ClinVar accession number SCV003230503.1 |
| E40 | F/11 mo | Focal seizures: turning the eyeballs to the side and upward, head turn to the right, since the age of 3 mo, focal EEG changes, low glucose in cerebrospinal fluid (glucose index 0.39); seizure-free after the introduction of ketogenic diet. |  | *SLC2A1* | c.982G>C,  (p.Val328Leu)/het/AD/de novo | GnomAD no frequency. This missense change has been observed in individual(s) with autosomal dominant GLUT1-deficiency(Gras et al., 2017). Advanced modeling of protein sequence and biophysical performed at Invitae indicates that this missense variant is expected to disrupt SLC2A1 protein function. ClinVar accession number SCV002223726.2 |
| E17 | M/8 y | Epilepsy with absence seizures, drug-resistant, since the age of 7 years. Complete seizure control on a ketogenic diet. Low glucose in cerebrospinal fluid. | In two sisters neurological problems. Older has myoclonic seizures at night, despite treatment with VIGA and lamotrigine. In younger sister tonic seizures since the age of 10 m, hypotonia, speech regression, mildly elevated lactic acid. One brother with migraine headaches. | *SLC2A1* | c.823G>A,  (p.Ala275Thr)/het/AD/maternal, the mother is healthy, variant segregates with epilepsy in maternal relatives (sister, sister`s son.), variant present in the older sister of the patient and one brother with migraine headaches. Absent in younger sister. | GnomAD no frequency). This missense change has been observed in individual(s) with SLC2A1-related conditions (Weber et al., 2008). Experimental studies have shown that this missense change affects SLC2A1 function (Weber et al., 2008).  ClinVar accession number SCV002236337.2 |
| E50 | F/18 mo | Focal seizures since the age of 3 days, started after vaccination, multiple seizures a day, seizures after each vaccination. Good response to carbamazepine. Normal speech development, mildly retarded motor development- crawling since the age of 13 mo, able to speak single words. |  | *KCNQ2* | c.587C>T, p.(Ala196Val)/het/  AD/de novo | GnomAD no frequency. This missense change has been observed in individual(s) with benign familial neonatal seizures and early onset epileptic encephalopathy(Soldovieri et al., 2007). Experimental studies have shown that this missense change affects KCNQ2 function (Soldovieri et al., 2007). ClinVar accession number SCV001231804.4 |
| E28 | M/12 mo | Born with a birthweight of 1760g at term, No congenital anomalies except for clinodactyly of the 4^th^ and 5^th^ fingers (the same in his mother). Profound developmental delay. At the age of 11 months was unable to sit. Myoclonic epilepsy, drug-resistant, with multiple episodes every day, started at the age of 8 mo, strabismus, spasticity, and absent speech. Bilateral interictal EEG changes. |  | *EEF1A2* | c.364G>A, p.(Glu122Lys)/het/  AD/de novo | The glutamic acid at position 122 is highly conserved ExAC no frequency. This variant has been reported to be de novo in multiple individuals affected with neurological disease, including epilepsy and intellectual disability (Nakajima et al., 2015).  Experimental studies have shown that this missense change disrupted the normal function of EEFIA2 to limit the frequency of errors during genetic code translation in yeast (Sandbaken & Culbertson, 1988). ClinVar accession number SCV000774988.4 |
| E55 | F/7 y | Focal epilepsy- since the age of 18 mo- eyelid myoclonus, episodic strabismus, good reaction to carbamazepine. At present absence seizures and clonic seizures of the right hand during the night. Normal MRI of the brain. |  | *NPRL3* | c.629+1G>T/het/AD/de novo/likely pathogenic | ExAC no frequency. This sequence change affects a donor splice site in intron 7 of the *NPRL3* gene. It is expected to disrupt RNA splicing. Disruption of this splice site has been observed in individual(s) with clinical features of *NPRL3*-related conditions (Invitae). ClinVar accession number SCV002274466.2 |
| E141 | M/11 y | Focal epilepsy, since the age of 4 y, drug-resistant. Seizures of the right half of the face, with hypersalivation, during sleep. Normal intellectual development, normal speech. Mild school problems. Normal MRI of the brain. |  | *GRIN2A* | c.1510C>T,  p.(Arg504Trp)/AD/NDA | GnomAD no frequency  This missense change has been observed in individuals with clinical features of GRIN2A-related conditions(Lesca et al., 2013). Experimental studies have shown that this missense change affects GRIN2A function (Swanger et al., 2016) ClinVar accession number SCV000946086.4 |
| E128 | F/13 mo | Pachygyria, subcortical heterotopia, enlarged ventricles, elevated lactic acid in urine and blood, deafness, focal epilepsy since infancy, no eye contact, severely delayed development. TANDEM MS normal. Array-CGH normal. |  | *DYNC1H1* | c.10016G>A,  (p.Arg3339His)/het/AD/de novo | GnomAD no frequency. This missense change has been observed in individual(s) with clinical features of DYNC1H1-related intellectual disability (Invitae). In at least one individual the variant was observed to be de novo. Advanced modeling of protein sequence and biophysical properties performed at Invitae indicates that this missense variant is expected to disrupt DYNC1H1 protein function. ClinVar accession number SCV002210260.2 |
| E104 | F/9 y | Seizures at the age of 2.5 years, during infection, second episode at the age of 5 years, also during infection. Last episode at the age of 9.5 years, during infection. Normal intellect, no dysmorphy, no other health problems. | Mother healthy | *SCN1B* | c.253C>T, p.(Arg85Cys)/het/AD/maternal | GnomAD no frequency  This missense change has been observed in individuals with autosomal dominant generalized epilepsy with febrile seizures plus (GEFS+) and autosomal recessive epileptic encephalopathy with cerebellar atrophy(Aeby et al., 2019; Scheffer et al., 2007). Algorithms developed to predict the effect of missense changes on protein structure and function are either unavailable or do not agree on the potential impact of this missense change (SIFT: "Deleterious"; PolyPhen-2: "Probably Damaging"; Align-GVGD: "Class C15"). Experimental studies have shown that this missense change affects SCN1B function (Xu et al., 2007). This variant disrupts the p.Arg85 amino acid residue in SCN1B. Other variant(s) that disrupt this residue have been determined to be pathogenic (Scheffer et al., 2007). ClinVar accession number SCV001407089.4 |
| E87 | F/9 y | Since the age of 3 years developmental delay, since the age of 5 years focal seizures. Persistent changes in EEG in the absence of clinical seizures. Mild intellectual disability, overweight. |  | *NEXMIF* | c.846_849del,  p.(Val283Thrfs*20, XL, NDA | This sequence change creates a premature translational stop signal in the NEXMIF gene. It is expected to result in an absent or disrupted protein product. Loss-of-function variants in NEXMIF are known to be pathogenic (PMID: 23615299). GnomAD no frequency. This premature translational stop signal has been observed in individual(s) with clinical features of NEXMIF-related conditions (Costain et al., 2019). ClinVar accession number SCV000931495.3 |
| E7 | M/43y | First seizure (tonic-clonic) at the age of 12 mo, provoked by infection, subsequent seizures not always related to fever. After the introduction of CARB worsening of clinical course with the appearance of tremors. Absent speech, severe intellectual disability. Dark hair, brown eyes. No microcephaly. |  | *UBE3A* | Deletion (Entire coding sequence) confirmed by MLPA/het/AD/de novo | A gross deletion of the genomic region encompassing the full coding sequence of the *UBE3A* gene |
| E43 | M/8y | Epilepsy with atonic seizures, focal seizures, and permanent EEG changes since the age of 16 mo. Intellectual disability, mild. Microcephaly. Pregnancy after AID. |  | *CHD2* | Deletion (Exons 2-28)/het/AD/NDA.  After array-CGH: deletion 15q26.1, 1.8Mbp | This variant is a gross deletion of the genomic region encompassing exon(s) 2-28 of the *CHD2* gene, which includes the initiator codon. Loss-of-function variants in *CHD2* are known to be pathogenic (Carvill et al., 2013). |
| E15 | F/6y | The phenotype of atypical Rett syndrome, with bruxism, lack of speech, focal epilepsy since age 2y, severe motor delay, and stereotypies. Motor development was impaired since birth. No hand apraxia. |  | *DDX3X* | c.1658_1661del  p.Thr553Argfs*18)/het/XL/de novo | It is expected to result in an absent or disrupted protein product. Loss-of-function variants in *DDX3X* are known to be pathogenic (Snijders Blok et al., 2015). GnomAD no frequency. ClinVar accession number SCV002237013.2 |
| E113 | M/12.5y | Epilepsy, with frequent focal seizures with impaired consciousness, lasting several hours. Normal intellect. ADHD. No dysmorphy, normal build. | Brother- epilepsy (same morphology of episodes), mother- epilepsy (same morphology) since the age of 5 years. Father- migraine with aura. | *CACNA1A* | c.5422G>A  (p.Ala1808Thr)/het/AD/maternal/the same variant in the patient`s brother  /likely pathogenic | GnomAD no frequency. This missense change has been observed in individuals with clinical features of CACNA1A-related conditions(Wang et al., 2021). Advanced modeling of protein sequence and biophysical properties performed at Invitae indicates that this missense variant is expected to disrupt CACNA1A protein function. ClinVar accession number SCV002196739.2 |
| E82 | F/9 y | Epilepsy, with focal seizures with hypersalivation and clonias of right leg and hand at the age of 5 mo and 18 mo. Episodes of sudden vomiting since the age of 3 mo, multiple episodes of acute ataxia, with dysarthria, vomiting, nystagmus, during up to 12 hours, 2-3 times a week. At present chronical mild ataxia, with wide-based gait, truncal hypotonia, dysdiadochokinesis, impaired speech. Normal MRI of the brain. |  | *SCN2A* | c.4972C>T, (p.Pro1658Ser)/het/AD/NDA | This sequence change replaces proline, which is neutral and non-polar, with serine, which is neutral and polar, at codon 1658 of the SCN2A  protein (p.Pro1658Ser).  This variant is not present in population databases (gnomAD no frequency).  This missense change has been observed in individuals with developmental epileptic encephalopathy (Miao et al., 2020). In at least one  individual the variant was observed to be de novo.  Advanced modeling of protein sequence and biophysical properties (such as structural, functional, and spatial information, amino acid  conservation, physicochemical variation, residue mobility, and thermodynamic stability) performed at Invitae indicates that this missense variant  is expected to disrupt SCN2A protein function.  Experimental studies have shown that this missense change affects SCN2A function (Miao et al., 2020) ClinVar accession number SCV001485395.3 |

AED- antiepileptic drugs, MRI- magnetic resonance imaging, AID- artificial insemination with sperm of donor, ADNFLE- autosomal dominant nocturnal frontal lobe epilepsy, ADHD- attention deficit hyperactivity disorder, mo- months, y-years, M-male, F-female, NDA- no data available, het- heterozygous, AD-autosomal dominant, XL- X-chromosome linked,

Aeby, A., Sculier, C., Bouza, A. A., Askar, B., Lederer, D., Schoonjans, A. S., Vander Ghinst, M., Ceulemans, B., Offord, J., Lopez-Santiago, L. F., & Isom, L. L. (2019). SCN1B-linked early infantile developmental and epileptic encephalopathy. *Annals of Clinical and Translational Neurology*, *6*(12). https://doi.org/10.1002/acn3.50921

Artuso, R., Mencarelli, M. A., Polli, R., Sartori, S., Ariani, F., Pollazzon, M., Marozza, A., Cilio, M. R., Specchio, N., Vigevano, F., Vecchi, M., Boniver, C., Dalla Bernardina, B., Parmeggiani, A., Buoni, S., Hayek, G., Mari, F., Renieri, A., & Murgia, A. (2010). Early-onset seizure variant of Rett syndrome: Definition of the clinical diagnostic criteria. *Brain and Development*, *32*(1). https://doi.org/10.1016/j.braindev.2009.02.004

Carvill, G. L., Heavin, S. B., Yendle, S. C., McMahon, J. M., O’Roak, B. J., Cook, J., Khan, A., Dorschner, M. O., Weaver, M., Calvert, S., Malone, S., Wallace, G., Stanley, T., Bye, A. M. E., Bleasel, A., Howell, K. B., Kivity, S., Mackay, M. T., Rodriguez-Casero, V., … Mefford, H. C. (2013). Targeted resequencing in epileptic encephalopathies identifies de novo mutations in CHD2 and SYNGAP1. *Nature Genetics*, *45*(7). https://doi.org/10.1038/ng.2646

Chen, W. J., Lin, Y., Xiong, Z. Q., Wei, W., Ni, W., Tan, G. H., Guo, S. L., He, J., Chen, Y. F., Zhang, Q. J., Li, H. F., Lin, Y., Murong, S. X., Xu, J., Wang, N., & Wu, Z. Y. (2011). Exome sequencing identifies truncating mutations in PRRT2 that cause paroxysmal kinesigenic dyskinesia. *Nature Genetics*, *43*(12). https://doi.org/10.1038/ng.1008

Costain, G., Cordeiro, D., Matviychuk, D., & Mercimek-Andrews, S. (2019). Clinical Application of Targeted Next-Generation Sequencing Panels and Whole Exome Sequencing in Childhood Epilepsy. *Neuroscience*, *418*. https://doi.org/10.1016/j.neuroscience.2019.08.016

Dale, R. C., Grattan-Smith, P., Nicholson, M., & Peters, G. B. (2012). Microdeletions detected using chromosome microarray in children with suspected genetic movement disorders: A single-centre study. *Developmental Medicine and Child Neurology*, *54*(7). https://doi.org/10.1111/j.1469-8749.2012.04287.x

Dlugos, D. J., Ferraro, T. N., & Buono, R. J. (2007). Novel De Novo Mutation of a Conserved SCN1A Amino-Acid Residue (R1596). *Pediatric Neurology*, *37*(4). https://doi.org/10.1016/j.pediatrneurol.2007.06.008

Fehr, S., Wilson, M., Downs, J., Williams, S., Murgia, A., Sartori, S., Vecchi, M., Ho, G., Polli, R., Psoni, S., Bao, X., De Klerk, N., Leonard, H., & Christodoulou, J. (2013). The CDKL5 disorder is an independent clinical entity associated with early-onset encephalopathy. *European Journal of Human Genetics*, *21*(3). https://doi.org/10.1038/ejhg.2012.156

Fukuma, G., Oguni, H., Shirasaka, Y., Watanabe, K., Miyajima, T., Yasumoto, S., Ohfu, M., Inoue, T., Watanachai, A., Kira, R., Matsuo, M., Muranaka, H., Sofue, F., Zhang, B., Kaneko, S., Mitsudome, A., & Hirose, S. (2004). Mutations of Neuronal Voltage-gated Na+ Channel α1 Subunit Gene SCN1A in Core Severe Myoclonic Epilepsy in Infancy (SMEI) and in Borderline SMEI (SMEB). *Epilepsia*, *45*(2). https://doi.org/10.1111/j.0013-9580.2004.15103.x

Gras, D., Cousin, C., Kappeler, C., Fung, C. W., Auvin, S., Essid, N., Chung, B. H., Da Costa, L., Hainque, E., Luton, M. P., Petit, V., Vuillaumier-Barrot, S., Boespflug-Tanguy, O., Roze, E., & Mochel, F. (2017). A simple blood test expedites the diagnosis of glucose transporter type 1 deficiency syndrome. *Annals of Neurology*, *82*(1). https://doi.org/10.1002/ana.24970

Harkin, L. A., McMahon, J. M., Iona, X., Dibbens, L., Pelekanos, J. T., Zuberi, S. M., Sadleir, L. G., Andermann, E., Gill, D., Farrell, K., Connolly, M., Stanley, T., Harbord, M., Andermann, F., Wang, J., Batish, S. D., Jones, J. G., Seltzer, W. K., Gardner, A., … Wirrell, E. (2007). The spectrum of SCN1A-related infantile epileptic encephalopathies. *Brain*, *130*(3). https://doi.org/10.1093/brain/awm002

Hız-Kurul, S., Gürsoy, S., Ayanoğlu, M., Yiş, U., & Erçal, D. (2017). Expanding spectrum of SCN1A-related phenotype with novel mutations. *Turkish Journal of Pediatrics*, *59*(5). https://doi.org/10.24953/turkjped.2017.05.010

Hoffman-Zacharska, D., Szczepanik, E., Terczynska, I., Goszczanska-Ciuchta, A., Zalewska-Miszkurka, Z., Tataj, R., & Bal, J. (2015). From focal epilepsy to dravet syndrome –heterogeneity of the phenotype due to SCN1A mutations of the p.Arg1596 amino acid residue in the nav1.1 subunit. *Neurologia i Neurochirurgia Polska*, *49*(4). https://doi.org/10.1016/j.pjnns.2015.06.006

Lesca, G., Rudolf, G., Bruneau, N., Lozovaya, N., Labalme, A., Boutry-Kryza, N., Salmi, M., Tsintsadze, T., Addis, L., Motte, J., Wright, S., Tsintsadze, V., Michel, A., Doummar, D., Lascelles, K., Strug, L., Waters, P., De Bellescize, J., Vrielynck, P., … Szepetowski, P. (2013). GRIN2A mutations in acquired epileptic aphasia and related childhood focal epilepsies and encephalopathies with speech and language dysfunction. *Nature Genetics*, *45*(9). https://doi.org/10.1038/ng.2726

Li, R., Fu, F., Yu, Q., Wang, D., Jing, X., Zhang, Y., Li, F., Li, F., Han, J., Pan, M., Zhen, L., Li, D., & Liao, C. (2020). Prenatal exome sequencing in fetuses with congenital heart defects. *Clinical Genetics*, *98*(3). https://doi.org/10.1111/cge.13774

Miao, P., Tang, S., Ye, J., Wang, J., Lou, Y., Zhang, B., Xu, X., Chen, X., Li, Y., & Feng, J. (2020). Electrophysiological features: The next precise step for SCN2A developmental epileptic encephalopathy. *Molecular Genetics and Genomic Medicine*, *8*(7). https://doi.org/10.1002/mgg3.1250

Nakajima, J., Okamoto, N., Tohyama, J., Kato, M., Arai, H., Funahashi, O., Tsurusaki, Y., Nakashima, M., Kawashima, H., Saitsu, H., Matsumoto, N., & Miyake, N. (2015). De novo EEF1A2 mutations in patients with characteristic facial features, intellectual disability, autistic behaviors and epilepsy. *Clinical Genetics*, *87*(4). https://doi.org/10.1111/cge.12394

Nemos, C., Lambert, L., Giuliano, F., Doray, B., Roubertie, A., Goldenberg, A., Delobel, B., Layet, V., N’guyen, M. A., Saunier, A., Verneau, F., Jonveaux, P., & Philippe, C. (2009). Mutational spectrum of CDKL5 in early-onset encephalopathies: A study of a large collection of French patients and review of the literature. *Clinical Genetics*, *76*(4). https://doi.org/10.1111/j.1399-0004.2009.01194.x

Picard, F., Makrythanasis, P., Navarro, V., Ishida, S., De Bellescize, J., Ville, D., Weckhuysen, S., Fosselle, E., Suls, A., De Jonghe, P., Raina, M. V., Lesca, G., Depienne, C., An-Gourfinkel, I., Vlaicu, M., Baulac, M., Mundwiller, E., Couarch, P., Combi, R., … Baulac, S. (2014). DEPDC5 mutations in families presenting as autosomal dominant nocturnal frontal lobe epilepsy. *Neurology*, *82*(23). https://doi.org/10.1212/WNL.0000000000000488

Sandbaken, M. G., & Culbertson, M. R. (1988). Mutations in elongation factor EF-1 alpha affect the frequency of frameshifting and amino acid misincorporation in Saccharomyces cerevisiae. *Genetics*, *120*(4). https://doi.org/10.1093/genetics/120.4.923

Scheffer, I. E., Harkin, L. A., Grinton, B. E., Dibbens, L. M., Turner, S. J., Zielinski, M. A., Xu, R., Jackson, G., Adams, J., Connellan, M., Petrou, S., Wellard, R. M., Briellmann, R. S., Wallace, R. H., Mulley, J. C., & Berkovic, S. F. (2007). Temporal lobe epilepsy and GEFS+ phenotypes associated with SCN1B mutations. *Brain*, *130*(1). https://doi.org/10.1093/brain/awl272

Snijders Blok, L., Madsen, E., Juusola, J., Gilissen, C., Baralle, D., Reijnders, M. R. F., Venselaar, H., Helsmoortel, C., Cho, M. T., Hoischen, A., Vissers, L. E. L. M., Koemans, T. S., Wissink-Lindhout, W., Eichler, E. E., Romano, C., Van Esch, H., Stumpel, C., Vreeburg, M., Smeets, E., … Kleefstra, T. (2015). Mutations in DDX3X Are a Common Cause of Unexplained Intellectual Disability with Gender-Specific Effects on Wnt Signaling. *American Journal of Human Genetics*, *97*(2). https://doi.org/10.1016/j.ajhg.2015.07.004

Soldovieri, M. V., Cilio, M. R., Miceli, F., Bellini, G., Del Giudice, E. M., Castaldo, P., Hernandez, C. C., Shapiro, M. S., Pascotto, A., Annunziato, L., & Taglialatela, M. (2007). Atypical gating of M-type potassium channels conferred by mutations in uncharged residues in the S4 region of KCNQ2 causing benign familial neonatal convulsions. *Journal of Neuroscience*, *27*(18). https://doi.org/10.1523/JNEUROSCI.0580-07.2007

Strizheva, G. D., Carsillo, T., Kruger, W. D., Sullivan, E. J., Ryu, J. H., & Henske, E. P. (2001). The spectrum of mutations in TSC1 and TSC2 in women with tuberous sclerosis and lymphangiomyomatosis. *American Journal of Respiratory and Critical Care Medicine*, *163*(1). https://doi.org/10.1164/ajrccm.163.1.2005004

Swanger, S. A., Chen, W., Wells, G., Burger, P. B., Tankovic, A., Bhattacharya, S., Strong, K. L., Hu, C., Kusumoto, H., Zhang, J., Adams, D. R., Millichap, J. J., Petrovski, S., Traynelis, S. F., & Yuan, H. (2016). Mechanistic Insight into NMDA Receptor Dysregulation by Rare Variants in the GluN2A and GluN2B Agonist Binding Domains. *American Journal of Human Genetics*, *99*(6). https://doi.org/10.1016/j.ajhg.2016.10.002

Verbeek, N. E., Van Kempen, M., Gunning, W. B., Renier, W. O., Westland, B., Lindhout, D., & Brilstra, E. H. (2011). Adults with a history of possible Dravet syndrome: An illustration of the importance of analysis of the SCN1A gene. *Epilepsia*, *52*(4). https://doi.org/10.1111/j.1528-1167.2011.02982.x

Volkers, L., Kahlig, K. M., Verbeek, N. E., Das, J. H. G., van Kempen, M. J. A., Stroink, H., Augustijn, P., van Nieuwenhuizen, O., Lindhout, D., George, A. L., Koeleman, B. P. C., & Rook, M. B. (2011). Na v1.1 dysfunction in genetic epilepsy with febrile seizures-plus or Dravet syndrome. *European Journal of Neuroscience*, *34*(8). https://doi.org/10.1111/j.1460-9568.2011.07826.x

Wang, T., Wa ng, J., Ma, Y., Zhou, H., Ding, D., Li, C., Du, X., Jiang, Y. H., Wang, Y., Long, S., Li, S., Lu, G., Chen, W., Zhou, Y., & Zhou, S. (2021). High genetic burden in 163 Chinese children with status epilepticus. *Seizure*, *84*. https://doi.org/10.1016/j.seizure.2020.10.032

Weber, Y. G., Storch, A., Wuttke, T. V., Brockmann, K., Kempfle, J., Maljevic, S., Margari, L., Kamm, C., Schneider, S. A., Huber, S. M., Pekrun, A., Roebling, R., Seebohm, G., Koka, S., Lang, C., Kraft, E., Blazevic, D., Salvo-Vargas, A., Fauler, M., … Lerche, H. (2008). GLUT1 mutations are a cause of paroxysmal exertion-induced dyskinesias and induce hemolytic anemia by a cation leak. *Journal of Clinical Investigation*, *118*(6). https://doi.org/10.1172/JCI34438

Xu, R., Thomas, E. A., Gazina, E. V., Richards, K. L., Quick, M., Wallace, R. H., Harkin, L. A., Heron, S. E., Berkovic, S. F., Scheffer, I. E., Mulley, J. C., & Petrou, S. (2007). Generalized epilepsy with febrile seizures plus-associated sodium channel β1 subunit mutations severely reduce beta subunit-mediated modulation of sodium channel function. *Neuroscience*, *148*(1). https://doi.org/10.1016/j.neuroscience.2007.05.038

Zuberi, S. M., Brunklaus, A., Birch, R., Reavey, E., Duncan, J., & Forbes, G. H. (2011). Genotype-phenotype associations in SCN1A-related epilepsies. *Neurology*, *76*(7). https://doi.org/10.1212/WNL.0b013e31820c309b
