# Supplementary material for: Validation of targeted next-generation sequencing panels in a cohort of Polish patients with epilepsy: assessing variable performance across clinical endophenotypes and uncovering novel genetic variants
Source: Front Neurol. 2024 Jan 12;14:1316933. doi: 10.3389/fneur.2023.1316933 (PMC10849089; doi:10.3389/fneur.2023.1316933)
Supplement: Supplementary file 2 [file Table_2.DOCX]

Supplement 2. List of genes in different panels analyzed in patients from our cohort.

1. **Panel with 104 genes (performed in one patient)**

ADSL, ALDH5A1, ALDH7A1, ARG1, ARSA, ARX, ASNS, ATRX, BRAT1, CACNA1A, CACNA1E, CAMK2B, CASK, CDKL5, CHD2, CLCN4, CLN2 (TPP1), CLN3, CLN5, CLN6, CLTC, CNTNAP2, CTNNB1, DDC, DDX3X, DEAF1, DNM1L, DYNC1H1, DYRK1A, EEF1A2, EHMT1, FOLR1, FOXG1, GABBR2, GABRB3, GABRG2, GAMT, GATAD2B, GATM, GNAO1, GRIA3, GRIN1, GRIN2A, GRIN2B, HEXA, HNRNPU, IQSEC2, KANSL1, KCNA2, KCNB1, KCNC1, KCNH1, KCNQ2, KCNT1, KIF1A, MBD5, MECP2, MEF2C, MFSD8, MTOR, NAGLU, NEXMIF, NGLY1, NPC1, NRXN1, PACS1, PACS2, PCDH19, POLG, PPP2R1A, PPP2R5D, PPP3CA, PPT1, PURA, QDPR, RAI1, SCN1A, SCN1B, SCN2A, SCN3A, SCN8A, SETBP1, SGSH, SLC13A5,, SLC2A1, SLC6A1, SLC6A8, SLC9A6, SMC1A, SPATA5, SPTAN1, STXBP1, SYNGAP1, TBCK, TBL1XR1, TCF4, TSC1, TSC2, UBE3A, UNC80, WDR45, WWOX, ZDHHC9, ZEB2.

1. **Panel with 150 genes (performed in one patient)**

ABAT, ADSL, ALDH5A1, ALDH7A1, AMT, ARG1, ARHGEF9, ARSA, ARX, ASAH1, ASNS, ATAD1, ATP1A3, ATP6AP2, ATP7A, ATRX, BRAT1, CACNA1A, CACNA1E, CAD, CAMK2B, CASK, CDKL5, CHD2, CLCN4, CLN2 (TPP1), CLN3, CLN5, CLN6, CLN8, CLTC, CNTNAP2, CTNNB1, CTSD, CYP27A1, DDC, DDX3X, DEAF1, DHFR, DNM1, DNM1L, DYNC1H1, DYRK1A, EEF1A2, EHMT1, FOLR1, FOXG1, FOXP1, GABBR2, GABRB3, GABRG2, GAMT, GATAD2B, GATM, GCH1, GLDC, GLRA1, GLRB, GNAO1, GPHN, GRIA3, GRIN1, GRIN2A, GRIN2B, HEXA, HNRNPU, IQSEC2, KANSL1, KCNA2, KCNB1, KCNH1, KCNJ10, KCNQ2, KCNT1, KCTD7, KIF1A, MBD5, MECP2, MEF2C, MFSD8, MOCS1, MOCS2, MTOR, NAGLU, NEXMIF, NGLY1, NPC1, NPC2, NRXN1, PACS1,PACS2, PCDH19, PEX10, PEX12, PEX13, PEX14, PEX16, PEX19, PEX2, PEX26, PEX3, PEX5, PEX6, PNPO, PHGDH, POLG, PP2R1A, PPP2R5D, PPP3CA, PPT1, PSAT1, PSPH, PTEN, PURA, QDPR, RAI1, SATB2, SCN1A, SCN1B, SCN2A, SCN3A, SCN8A, SETBP1, SGSH, SLC13A5, SLC19A3, SLC25A12, SLC2A1, SLC6A1, SLC6A8, SLC9A6, SMC1A, SPATA5, SPTAN1, STXBP1, SURF1, SYNGAP1, TBCK, TBL1XR1, TCF4, TH, TPK1, TSC1, TSC2, UBE3A, UNC80, WDR45, WWOX, ZDHHC9, ZEB2

1. **Panel with 181 genes (performed in 3 patients)**

Invitae Epilepsy Panel (146)

ADSL, ALDH5A1, ALDH7A1, ALG13, ARHGEF9, ARX, ATP1A2, ATP1A3, ATRX, BRAT1, C12orf57, CACNA1A, CACNA2D2, CARS2, CASK, CDKL5, CHD2, CHRNA2, CHRNA4, CHRNB2, CLCN4, CLN2 (TPP1), CLN3, CLN5, CLN6, CLN8, CNTNAP2, CSTB, CTSD, DEPDC5, DNAJC5, DNM1, DOCK7, DYRK1A, EEF1A2, EFHC1, EHMT1, EPM2A, FARS2, FOLR1, FOXG1, FRRS1L, GABBR2, GABRA1, GABRB2, GABRB3, GABRG2, GAMT, GATM, GLRA1, GNAO1, GOSR2, GRIN1, GRIN2A, GRIN2B, HCN1, HNRNPU, IER3IP1, IQSEC2, ITPA, JMJD1C, KANSL1, KCNA2, KCNB1, KCNC1, KCNH2, KCNJ10, KCNMA1, KCNQ2, KCNQ3, KCNT1, KCTD7, LGI1, LIAS, MBD5, MECP2, MEF2C, MFSD8, MTOR, NEDD4L, NEXMIF, NGLY1, NHLRC1, NPRL3, NRXN1, PACS1, PCDH19, PIGA, PIGN, PIGO, PLCB1, PNKD, PNKP, PNPO, POLG, PPT1, PRICKLE1, PRIMA1, PRRT2, PURA, QARS, RELN, ROGDI, SATB2, SCARB2, SCN1A, SCN1B, SCN2A, SCN3A, SCN8A, SCN9A, SERPINI1, SGCE, SIK1, SLC12A5, SLC13A5, SLC19A3, SLC25A12, SLC25A22, SLC2A1, SLC35A2, SLC6A1, SLC6A8, SLC9A6, SMC1A, SNX27, SPATA5, SPTAN1, ST3GAL5, STRADA, STX1B, STXBP1, SYN1, SYNGAP1, SYNJ1, SZT2, TBC1D24, TCF4, TPK1, TSC1, TSC2, UBE3A, WDR45, WWOX, ZDHHC9, ZEB2

Add-on Preliminary-evidence Genes for Epilepsy (35)

ABAT, ARHGEF15, ATP6AP2, CACNA1H, CACNB4, CASR, CERS1, CNTN2, CPA6, DIAPH1, FASN, GABRD, GAL, GPHN, KCNA1, KCND2, KCNH5, KPNA7, LMNB2, NECAP1, PIGG, PIGQ, PIK3AP1, PRDM8, PRICKLE2, RBFOX1, RBFOX3, RYR3, SCN5A, SETD2, SLC35A3, SNAP25, SRPX2, ST3GAL3, TBL1XR1

1. **Panel with 184 genes (performed in 4 patients)**

Invitae Epilepsy Panel (146)

ADSL, ALDH5A1, ALDH7A1, ALG13, ARHGEF9, ARX, ATP1A2, ATP1A3, ATRX, BRAT1, C12orf57, CACNA1A, CACNA2D2, CARS2, CASK, CDKL5, CHD2, CHRNA2, CHRNA4, CHRNB2, CLCN4, CLN2 (TPP1), CLN3, CLN5, CLN6, CLN8, CNTNAP2, CSTB, CTSD, DEPDC5, DNAJC5, DNM1, DOCK7, DYRK1A, EEF1A2, EFHC1, EHMT1, EPM2A, FARS2, FOLR1, FOXG1, FRRS1L, GABBR2, GABRA1, GABRB2, GABRB3, GABRG2, GAMT, GATM, GLRA1, GNAO1, GOSR2, GRIN1, GRIN2A, GRIN2B, HCN1, HNRNPU, IER3IP1, IQSEC2, ITPA, JMJD1C, KANSL1, KCNA2, KCNB1, KCNC1, KCNH2, KCNJ10, KCNMA1, KCNQ2, KCNQ3, KCNT1, KCTD7, LGI1, LIAS, MBD5, MECP2, MEF2C, MFSD8, MTOR, NEDD4L, NEXMIF, NGLY1, NHLRC1, NPRL3, NRXN1, PACS1, PCDH19, PIGA, PIGN, PIGO, PLCB1, PNKD, PNKP, PNPO, POLG, PPT1, PRICKLE1, PRIMA1, PRRT2, PURA, QARS, RELN, ROGDI, SATB2, SCARB2, SCN1A, SCN1B, SCN2A, SCN3A, SCN8A, SCN9A, SERPINI1, SGCE, SIK1, SLC12A5, SLC13A5, SLC19A3, SLC25A12, SLC25A22, SLC2A1, SLC35A2, SLC6A1, SLC6A8, SLC9A6, SMC1A, SNX27, SPATA5, SPTAN1, ST3GAL5, STRADA, STX1B, STXBP1, SYN1, SYNGAP1, SYNJ1, SZT2, TBC1D24, TCF4, TPK1, TSC1, TSC2, UBE3A, WDR45, WWOX, ZDHHC9, ZEB2

Add-on Preliminary-evidence Genes for Epilepsy (35)

ABAT, ARHGEF15, ATP6AP2, CACNA1H, CACNB4, CASR, CERS1, CNTN2, CPA6, DIAPH1, FASN, GABRD, GAL, GPHN, KCNA1, KCND2, KCNH5, KPNA7, LMNB2, NECAP1, PIGG, PIGQ, PIK3AP1, PRDM8, PRICKLE2, RBFOX1, RBFOX3, RYR3, SCN5A, SETD2, SLC35A3, SNAP25, SRPX2, ST3GAL3, TBL1XR1

Add-on Genes for Glycine Encephalopathy (3)

AMT, GCSH, GLDC

1. **Panel with 186 genes (performed in 7 patients)**

Invitae Epilepsy Panel (151)

ADSL, ALDH5A1, ALDH7A1, ALG13, ARG1, ARHGEF9, ARX, ATP1A2, ATP1A3, ATRX, BRAT1, C12orf57, CACNA1A, CACNA2D2, CARS2, CASK, CDKL5, CHD2, CHRNA2, CHRNA4, CHRNB2, CLCN4, CLN2 (TPP1), CLN3, CLN5, CLN6, CLN8, CNTNAP2, CSTB, CTSD, DDC, DEPDC5, DNAJC5, DNM1, DOCK7, DYRK1A, EEF1A2, EFHC1, EHMT1, EPM2A, FARS2, FOLR1, FOXG1, FRRS1L, GABBR2, GABRA1, GABRB2, GABRB3, GABRG2, GAMT, GATM, GLRA1, GNAO1, GOSR2, GRIN1, GRIN2A, GRIN2B, HCN1, HNRNPU, IER3IP1, IQSEC2, ITPA, JMJD1C, KANSL1, KCNA2, KCNB1, KCNC1, KCNH2, KCNJ10, KCNMA1, KCNQ2, KCNQ3, KCNT1, KCTD7, LGI1, LIAS, MBD5, MECP2, MEF2C, MFSD8, MOCS1, MOCS2, MTOR, NEDD4L, NEXMIF, NGLY1, NHLRC1, NPRL3, NRXN1, PACS1, PCDH19, PIGA, PIGN, PIGO, PLCB1, PNKD, PNKP, PNPO, POLG, PPT1, PRICKLE1, PRIMA1, PRRT2, PURA, QARS, RELN, ROGDI, SATB2, SCARB2, SCN1A, SCN1B, SCN2A, SCN3A, SCN8A, SCN9A, SERPINI1, SGCE, SIK1, SLC12A5, SLC13A5, SLC19A3, SLC25A12, SLC25A22, SLC2A1, SLC35A2, SLC6A1, SLC6A8, SLC9A6, SMC1A, SNX27, SPATA5, SPTAN1, ST3GAL5, STRADA, STX1B, STXBP1, SUOX, SYN1, SYNGAP1, SYNJ1, SZT2, TBC1D24, TCF4, TPK1, TSC1, TSC2, UBE3A, WDR45, WWOX, ZDHHC9, ZEB2

Add-on Preliminary-evidence Genes for Epilepsy (35)

ABAT, ARHGEF15, ATP6AP2, CACNA1H, CACNB4, CASR, CERS1, CNTN2, CPA6, DIAPH1, FASN, GABRD, GAL, GPHN, KCNA1, KCND2, KCNH5, KPNA7, LMNB2, NECAP1, PIGG, PIGQ, PIK3AP1, PRDM8, PRICKLE2, RBFOX1, RBFOX3, RYR3, SCN5A, SETD2, SLC35A3, SNAP25, SRPX2, ST3GAL3, TBL1XR1

1. **Invitae Epilepsy Panel (290)- performed in 35 patients**

AARS, ABAT, ADAR, ADSL, ALDH5A1, ALDH7A1, ALG1, ALG12, ALG13, ALG6, AMACR, AMT, AP2M1, AP3B2, ARG1, ARHGEF9, ARSA, ARX, ASAH1, ASNS, ATAD1, ATP1A2, ATP1A3, ATP6AP2, ATRX, BRAT1, C12orf57, CACNA1A, CACNA1E, CACNA2D2, CAD, CAMK2B, CARS2, CASK, CCDC88A, CDKL5, CHD2, CHRNA2, CHRNA4, CHRNB2, CLCN4, CLCN6, CLN2 (TPP1), CLN3, CLN5, CLN6, CLN8, CLTC, CNTN2, CNTNAP2, COG5, COL18A1, CSTB, CTNNB1, CTSD, CYFIP2, CYP27A1, DDC, DDX3X, DEAF1, DEPDC5, DHDDS, DHFR, DIAPH1, DNAJC5, DNM1, DNM1L, DOCK7, DYNC1H1, DYRK1A, ECHS1, EEF1A2, EHMT1, EMC1, EPM2A, FAR1, FARS2, FBXO11, FGF12, FLNA, FOLR1, FOXG1, FRRS1L, GABBR2, GABRA1, GABRB1, GABRB2, GABRB3, GABRG2, GAMT, GATAD2B, GATM, GCH1, GLDC, GLRA1, GLRB, GNAO1, GNB1, GOSR2, GPAA1, GPHN, GRIA3, GRIN1, GRIN2A, GRIN2B, GRIN2D, GTPBP3, HCN1, HDAC8, HEXA, HNRNPU, IER3IP1, IFIH1, IQSEC2, ITPA, KANSL1, KCNA1, KCNA2, KCNB1, KCNC1, KCND2, KCNH1, KCNH2, KCNH5, KCNJ10, KCNK4, KCNMA1, KCNQ2, KCNQ3, KCNQ5, KCNT1, KCTD7, KIF1A, KIF2A, KIF5A, KPNA7, LAMC3, LGI1, LIAS, MBD5, MDH2, MECP2, MEF2C, MFSD8, MOCS1, MOCS2, MTOR, NACC1, NAGLU, NECAP1, NEDD4L, NEXMIF, NGLY1, NHLRC1, NPC1, NPC2, NPRL3, NRXN1, NTRK2, NUS1, PACS1, PACS2, PAFAH1B1, PCDH19, PCLO, PEX10, PEX12, PEX13, PEX14, PEX16, PEX19, PEX2, PEX26, PEX3, PEX5, PEX6, PHGDH, PIGA, PIGG, PIGN, PIGO, PIGP, PIGQ, PIGV, PIGW, PLAA, PLCB1, PNKD, PNKP, PNPO, PNPT1, POLG, PPP2CA, PPP2R1A, PPP2R5D, PPP3CA, PPT1, PRICKLE1, PRIMA1, PRRT2, PSAP, PSAT1, PSPH, PTEN, PTPN23, PURA, QARS, QDPR, RAB11A, RAB11B, RAI1, RALA, RANBP2, RELN, RFT1, RHOBTB2, RNASEH2A, RNASEH2B, RNASEH2C, RNF13, ROGDI, RORB, RUSC2, SAMHD1, SATB2, SCARB2, SCN1A, SCN1B, SCN2A, SCN3A, SCN8A, SCN9A, SCP2, SERPINI1, SETBP1, SGCE, SGSH, SIK1, SLC12A5, SLC13A5, SLC19A3, SLC1A2, SLC25A12, SLC25A22, SLC2A1, SLC35A2, SLC6A1, SLC6A5, SLC6A8, SLC9A6, SMC1A, SNAP25, SNX27, SPATA5, SPTAN1, ST3GAL3, ST3GAL5, STAG2, STRADA, STX1B, STXBP1, STXBP2, SUMF1, SUOX, SYN1, SYNGAP1, SYNJ1, SZT2, TANGO2, TBC1D24, TBCK, TBL1XR1, TCF4, TH, TK2, TPK1, TREX1, TSC1, TSC2, TSFM, TUBB2A, UBA5, UBE3A, UNC80, WDR45, WWOX, YWHAG, ZDHHC9, ZEB2, ZSWIM6

1. **Panel with 302 genes- performed in 66 patients**

Invitae Epilepsy Panel (302)

AARS, ABAT, ADAR, ADSL, ALDH5A1, ALDH7A1, ALG1, ALG12, ALG13, ALG6, AMACR, AMT, AP2M1, AP3B2, ARG1, ARHGEF9, ARSA, ARX, ASAH1, ASNS, ATAD1, ATP1A1, ATP1A2, ATP1A3, ATP6AP2, ATP7A, ATRX, BRAT1, C12orf57, CACNA1A, CACNA1B, CACNA1E, CACNA2D2, CAD, CAMK2B, CARS2, CASK, CCDC88A, CDKL5, CHD2, CHRNA2, CHRNA4, CHRNB2, CLCN4, CLCN6, CLN2 (TPP1), CLN3, CLN5, CLN6, CLN8, CLTC, CNTN2, CNTNAP2, COG5, COL18A1, CSTB, CTNNB1, CTSD, CYFIP2, CYP27A1, DDC, DDX3X, DEAF1, DENND5A, DEPDC5, DHDDS, DHFR, DIAPH1, DMXL2, DNAJC5, DNM1, DNM1L, DOCK7, DYNC1H1, DYRK1A, ECHS1, EEF1A2, EHMT1, EMC1, EPM2A, FAR1, FARS2, FBXO11, FGF12, FLNA, FOLR1, FOXG1, FOXP1, FRRS1L, GABBR2, GABRA1, GABRA2, GABRB1, GABRB2, GABRB3, GABRG2, GAMT, GATAD2B, GATM, GCH1, GLDC, GLRA1, GLRB, GNAO1, GNB1, GOSR2, GPAA1, GPHN, GRIA3, GRIN1, GRIN2A, GRIN2B, GRIN2D, GTPBP3, HCN1, HDAC8, HEXA, HNRNPU, IER3IP1, IFIH1, IQSEC2, ITPA, KANSL1, KCNA1, KCNA2, KCNB1, KCNC1, KCND2, KCNH1, KCNH2, KCNH5, KCNJ10, KCNK4, KCNMA1, KCNQ2, KCNQ3, KCNQ5, KCNT1, KCTD7, KIF1A, KIF2A, KIF5A, KPNA7, LAMC3, LGI1, LIAS, MBD5, MDH2, MECP2, MEF2C, MFSD8, MICAL1, MOCS1, MOCS2, MTOR, NACC1, NAGLU, NECAP1, NEDD4L, NEXMIF, NGLY1, NHLRC1, NPC1, NPC2, NPRL3, NRXN1, NTRK2, NUS1, PACS1, PACS2, PAFAH1B1, PCDH19, PCLO, PEX10, PEX12, PEX13, PEX14, PEX16, PEX19, PEX2, PEX26, PEX3, PEX5, PEX6, PHGDH, PIGA, PIGB, PIGG, PIGN, PIGO, PIGP, PIGQ, PIGV, PIGW, PLAA, PLCB1, PNKD, PNKP, PNPO, PNPT1, POLG, PPP2CA, PPP2R1A, PPP2R5D, PPP3CA, PPT1, PRICKLE1, PRIMA1, PROSC, PRRT2, PSAP, PSAT1, PSPH, PTEN, PTPN23, PURA, QARS, QDPR, RAB11A, RAB11B, RAI1, RALA, RANBP2, RELN, RFT1, RHOBTB2, RNASEH2A, RNASEH2B, RNASEH2C, RNF13, ROGDI, RORB, RUSC2, SAMHD1, SATB2, SCARB2, SCN1A, SCN1B, SCN2A, SCN3A, SCN8A, SCN9A, SCP2, SERPINI1, SETBP1, SGCE, SGSH, SIK1, SLC12A5, SLC13A5, SLC19A3, SLC1A2, SLC25A12, SLC25A22, SLC2A1, SLC35A2, SLC6A1, SLC6A5, SLC6A8, SLC9A6, SMC1A, SNAP25, SNX27, SPATA5, SPTAN1, ST3GAL3, ST3GAL5, STAG2, STRADA, STX1B, STXBP1, STXBP2, SUMF1, SUOX, SURF1, SYN1, SYNGAP1, SYNJ1, SZT2, TANGO2, TBC1D24, TBCK, TBL1XR1, TCF4, TH, TK2, TPK1, TREX1, TRIM8, TSC1, TSC2, TSFM, TUBB2A, UBA5, UBE3A, UNC80, WDR45, WWOX, YWHAG, ZDHHC9, ZEB2, ZSWIM6

1. **Panel with 304 genes- performed in 21 patients**

Invitae Epilepsy Panel (304)

AARS, ABAT, ADAR, ADSL, ALDH5A1, ALDH7A1, ALG1, ALG12, ALG13, ALG6, AMACR, AMT, AP2M1, AP3B2, ARG1, ARHGEF15, ARHGEF9, ARSA, ARX, ASAH1, ASNS, ATAD1, ATP1A2, ATP1A3, ATP6AP2, ATRX, BRAT1, C12orf57, CACNA1A, CACNA1E, CACNA1H, CACNA2D2, CAD, CAMK2B, CARS2, CASK, CCDC88A, CDKL5, CERS1, CHD2, CHRNA2, CHRNA4, CHRNB2, CLCN4, CLCN6, CLN2 (TPP1), CLN3, CLN5, CLN6, CLN8, CLTC, CNTN2, CNTNAP2, COG5, COL18A1, CSTB, CTNNB1, CTSD, CYFIP2, CYP27A1, DDC, DDX3X, DEAF1, DEPDC5, DHDDS, DHFR, DIAPH1, DNAJC5, DNM1, DNM1L, DOCK7, DYNC1H1, DYRK1A, ECHS1, EEF1A2, EHMT1, EMC1, EPM2A, FAR1, FARS2, FASN, FBXO11, FGF12, FOLR1, FOXG1, FRRS1L, GABBR2, GABRA1, GABRB1, GABRB2, GABRB3, GABRG2, GAMT, GATAD2B, GATM, GCH1, GLDC, GLRA1, GLRB, GNAO1, GNB1, GOSR2, GPAA1, GPHN, GRIA3, GRIN1, GRIN2A, GRIN2B, GRIN2D, GTPBP3, GUF1, HCN1, HEXA, HNRNPU, HTT, IDH3A, IER3IP1, IFIH1, IQSEC2, ITPA, KANSL1, KCNA1, KCNA2, KCNB1, KCNC1, KCND2, KCNH1, KCNH2, KCNH5, KCNJ10, KCNK4, KCNMA1, KCNQ2, KCNQ3, KCNQ5, KCNT1, KCTD7, KIF1A, KIF2A, KIF5A, KPNA7, LAMC3, LGI1, LIAS, LMNB2, MBD5, MDH2, MECP2, MEF2C, MFSD8, MOCS1, MOCS2, MOCS3, MTOR, NACC1, NAGLU, NECAP1, NEDD4L, NEXMIF, NGLY1, NHLRC1, NPC1, NPC2, NPRL3, NRXN1, NTRK2, NUS1, PACS1, PACS2, PAFAH1B1, PCDH19, PCLO, PEX10, PEX12, PEX13, PEX14, PEX16, PEX19, PEX2, PEX26, PEX3, PEX5, PEX6, PHGDH, PIGA, PIGG, PIGN, PIGO, PIGP, PIGQ, PIGV, PIGW, PIK3AP1, PLAA, PLCB1, PNKD, PNKP, PNPO, PNPT1, POLG, PPP2CA, PPP2R1A, PPP2R5D, PPP3CA, PPT1, PRDM8, PRICKLE1, PRICKLE2, PRIMA1, PRRT2, PSAP, PSAT1, PSPH, PTPN23, PURA, QARS, QDPR, RAB11A, RAB11B, RAI1, RALA, RANBP2, RBFOX1, RBFOX3, RELN, RFT1, RHOBTB2, RNASEH2A, RNASEH2B, RNASEH2C, RNF13, ROGDI, RORB, RUSC2, SAMHD1, SATB2, SCARB2, SCN1A, SCN1B, SCN2A, SCN3A, SCN5A, SCN8A, SCN9A, SCP2, SERPINI1, SETBP1, SGCE, SGSH, SIK1, SLC12A5, SLC13A5, SLC19A3, SLC1A2, SLC25A12, SLC25A22, SLC2A1, SLC35A2, SLC6A1, SLC6A5, SLC6A8, SLC9A6, SMC1A, SNAP25, SNIP1, SNX27, SPATA5, SPTAN1, ST3GAL3, ST3GAL5, STAG2, STRADA, STX1B, STXBP1, STXBP2, SUMF1, SUOX, SYN1, SYNGAP1, SYNJ1, SZT2, TANGO2, TBC1D24, TBCK, TBL1XR1, TCF4, TH, TK2, TPK1, TREX1, TSC1, TSC2, TSFM, TUBA8, TUBB2A, UBA5, UBE3A, UNC80, WDR45, WWOX, YWHAG, ZDHHC9, ZEB2, ZSWIM6
